# Whole-Genome Analysis of Multienvironment or Multitrait QTL in MAGIC

**DOI:** 10.1534/g3.114.012971

**Published:** 2014-09-01

**Authors:** Arūnas P. Verbyla, Colin R. Cavanagh, Klara L. Verbyla

**Affiliations:** * Computational Informatics and Food Futures National Research Flagship, CSIRO, Atherton, QLD 4883, Australia; † School of Agriculture, Food and Wine, The University of Adelaide, Adelaide, SA 5005, Australia; ‡ Plant Industry and Food Futures National Research Flagship, CSIRO, Canberra, ACT 2601, Australia; § Computational Informatics and Food Futures National Research Flagship, CSIRO, Canberra, ACT 2601, Australia

**Keywords:** mixed models, multienvironment, multitrait, QTL, WGAIM, Multiparent Advanced Generation Inter-Cross (MAGIC), multiparental populations, MPP

## Abstract

Multiparent Advanced Generation Inter-Cross (MAGIC) populations are now being utilized to more accurately identify the underlying genetic basis of quantitative traits through quantitative trait loci (QTL) analyses and subsequent gene discovery. The expanded genetic diversity present in such populations and the amplified number of recombination events mean that QTL can be identified at a higher resolution. Most QTL analyses are conducted separately for each trait within a single environment. Separate analysis does not take advantage of the underlying correlation structure found in multienvironment or multitrait data. By using this information in a joint analysis—be it multienvironment or multitrait — it is possible to gain a greater understanding of genotype- or QTL-by-environment interactions or of pleiotropic effects across traits. Furthermore, this can result in improvements in accuracy for a range of traits or in a specific target environment and can influence selection decisions. Data derived from MAGIC populations allow for founder probabilities of all founder alleles to be calculated for each individual within the population. This presents an additional layer of complexity and information that can be utilized to identify QTL. A whole-genome approach is proposed for multienvironment and multitrait QTL analysis in MAGIC. The whole-genome approach simultaneously incorporates all founder probabilities at each marker for all individuals in the analysis, rather than using a genome scan. A dimension reduction technique is implemented, which allows for high-dimensional genetic data. For each QTL identified, sizes of effects for each founder allele, the percentage of genetic variance explained, and a score to reflect the strength of the QTL are found. The approach was demonstrated to perform well in a small simulation study and for two experiments, using a wheat MAGIC population.

DISCOVERING the underlying genes affecting important traits such as yield, quality, disease resistance, and climate adaptability is of paramount importance to increase the agricultural productivity needed to feed the world’s growing population. The first stage in identifying these genes is QTL analyses. Traditionally in plants, biparental crosses are used to create experimental populations on which QTL analyses were carried out. Recently the advantages of a different type of experimental cross, the Multiparent Advanced Generation Inter-Cross (MAGIC), have been explored. One of the most compelling reasons for developing MAGIC populations is the ability to conduct research on a broad range of traits in genetically diversity populations. The genetic diversity present in MAGIC populations is maximized through the selection of multiple genetically diverse founders. The founder lines in a classic plant MAGIC population are intercrossed until all founders have an equal probability of contributing to the genetic makeup of a line (more complex intercrossing patterns can be used). In plants this initial intercross is followed by multiple generations of selfing to create recombinant inbred lines (RILs). Such a structure leads to an amplified number of recombination events, which means that any QTL can be mapped at a higher resolution. Consequently, these populations are now being utilized to more accurately identify the underlying genetic basis of quantitative traits through quantitative trait loci (QTL) analyses (Mott *et al.* 2000; Cavanagh *et al.* 2008; Trebbi *et al.* 2008; Kover *et al.* 2009; Huang *et al.* 2012; Bandillo *et al.* 2013).

One point of difference between the QTL approaches that have been used is whether they use marker scores or founder probabilities. The structure of MAGIC populations means that the probabilities that an allele has been inherited from each founder can be calculated. These probabilities can contain additional information as the marker scores may not be fully informative in a population with multiple founders. Xu (1996) described the first QTL analyses for a MAGIC population, using marker scores with interval mapping to analyze a four-way cross. Contrastingly, Mott *et al.* (2000) found that using marker scores failed and described the first use of founder probabilities in QTL analyses for MAGIC. Other methods have used probabilities rather than marker scores (Kover *et al.* 2009; Huang *et al.* 2012; King *et al.* 2012; Verbyla *et al.* 2014).

Regardless of whether marker scores or probabilities are used, most studies have employed QTL approaches that use genome scans to test each marker or interval separately for association or linkage with the trait of interest (Xu 1996; Mott *et al.* 2000; Kover *et al.* 2009; Malosetti *et al.* 2011; King *et al.* 2012). An alternative is to use all information simultaneously in a single model, overcoming the need for genome scans. Whole-genome average interval mapping (WGAIM) was proposed by Verbyla *et al.* (2007) for biparental populations and modified by Verbyla *et al.* (2012). WGAIM was shown to outperform composite-interval mapping (CIM). The approach allows for population structure to be modeled and for any nongenetic effects, such as experimental design terms, to be easily included. A likelihood-ratio test of significance is conducted to decide whether selection of a putative QTL is warranted. Forward selection of putative QTL continues until the likelihood-ratio test is nonsignificant. WGAIM was extended for use in MAGIC populations by Verbyla *et al.* (2014), utilizing the probabilities of inheriting founder alleles for each individual at each locus.

To date, most QTL analyses in MAGIC are conducted separately for each trait within a single environment. However, separate QTL analyses (for any population) do not take advantage of the underlying correlation structure found in multienvironment or multitrait data. Joint analyses provide the opportunity for a greater understanding of genotype- or QTL-by-environment interactions or of pleiotropic effects across traits. This can result in a greater understanding of traits or environments and the relationship between them. This information could lead to improvements in accuracy for a range of traits or in a specific target environment and can influence selection decisions.

In biparental populations, the advantages presented by multivariate approaches have led to the development of a range of approaches for the analysis of multienvironment trials for a single trait (Jiang and Zeng 1995; Tinker and Mather 1995; Wang *et al.* 1999; Piepho 2000; Verbyla *et al.* 2003; Vargas *et al.* 2006; Boer *et al.* 2007). In addition, multitrait analysis has also been considered by many authors (Korol *et al.* 1995, 1998; Zeng *et al.* 1999; Knott and Haley 2000; Gilbert and Le Roy 2003; Lund *et al.* 2003). Hackett *et al.* (2001) present a review and an interval-mapping method based on multivariate regression. A more recent review of methods for multienvironment QTL analysis is presented by Van Eeuwijk *et al.* (2010). Malosetti *et al.* (2008) investigate multitrait, multienvironment analysis. An approach for multivariate QTL analysis in biparental populations based on WGAIM was presented by Verbyla and Cullis (2012).

In this article, an approach for multivariate QTL analysis is proposed for MAGIC populations, building upon Verbyla and Cullis (2012) and Verbyla *et al.* (2014); the approach is called multivariate multiparent (MVMP)WGAIM. Multivariate QTL effects are included in the model for all intervals (or markers) on the linkage map simultaneously. Probabilities of inheriting founder alleles are an integral part of the model and allow estimation of QTL sizes for all founder alleles. These multivariate genetic QTL sizes are modeled as random effects with an associated variance–covariance matrix, be they traits or environments. A likelihood-ratio test is presented for testing the significance of the QTL variance–covariance matrix. If the test is significant, a multivariate outlier detection method is used to select the most likely interval for a QTL. Multivariate QTL are chosen in a forward selection process. These QTL are also included in the random effects. For multienvironment QTL analysis it is possible that the QTL effects are the same over all environments. A test for the interaction of QTL by environment can be carried out by including a main effect QTL. The final summary of QTL effects includes a level of significance, a score that indicates the strength of the QTL, and the percentage of genetic variance accounted for by each QTL. A small simulation study examines type I error rates and power of the approach, complemented by multitrait and multienvironment examples using wheat MAGIC data, presented for illustration and interpretation.

## Methods

The approach presented generalizes that presented for the univariate (single trait or environment) situation (Verbyla *et al.* 2014). The methods presented are largely self-contained, however, even though the development matches the univariate case as given by Verbyla *et al.* (2014).

### Base model

In the plant sciences, genetic studies are usually based on designed experiments, often multiphase in nature (Smith *et al.* 2006). To provide a statistical analysis for these experiments, a linear mixed model is often used. This model has the form

$$y=X\tau +Z_{\text{o}}u_{\text{o}}+Z_{\text{g}}u_{\text{g}}+e,$$ (1)

where **y** is the vector of responses, which may comprise multiple traits or a single trait scored in multiple environments. The fixed effects **Xτ** and random effects **Z**_o_**u**_o_ reflect the experimental design and nongenetic effects while the residual vector **e** allows, for example, for possible spatial trends within environments or relationships between traits. Note that it is assumed **u**_o_ ∼ *N*(**0**, **G**_o_) and **e** ∼ *N*(**0**, **R**) and that they are uncorrelated. The genetic effects **Z**_g_**u**_g_ are discussed in the following section.

### Genetic model

Suppose there are *n*_g_ lines or varieties and *t* traits or environments in the study. The *n*_g_*t* × 1 vector of genetic effects is given by **u**_g_ in (1) and the matrix **Z**_g_ assigns the appropriate line to each observation in **y**. Without marker data, models for **u**_g_ are typically based on the infinitesimal or polygenic model. We consider the simplest polygenic model

$$u_{\text{g}}=u_{\text{p}},$$ (2)

where **u**_p_ is the polygenic effect assumed, $u_{\text{p}}∼N\left(0,G_{\text{p}}⊗I_{n_{\text{g}}}\right)$, where **G**_p_ is the *t* × *t* genetic variance–covariance matrix between environments or traits. This model can be extended to include a relationship matrix based on pedigree information.

The aim of this article is to use marker information in the determination of QTL for a MAGIC population. Suppose there are *n*_f_ founders in the MAGIC population and that we have *c* linkage groups (or chromosomes) and *r_k_* markers on linkage group *k*, *k* = 1, 2, … , *c*. We allow for a QTL in every interval or at each marker if analysis is based on markers; the development assumes intervals rather than markers but the marker-based analog simply replaces *r_k_* − 1 by *r_k_* where appropriate. Our model for the genetic effect for line *i* for trait or environment *s*, *u_gis_*, is given by

$$u_{gis}=\sum_{k=1}^{c}\sum_{j=1}^{r_{k}-1}q_{ikj}^{T}a_{kjs}+u_{pis},$$

where **q***_ikj_* is the *n*_f_ × 1 vector that indicates the founder allele for line *i* for a potential QTL in interval *j* on linkage group *k*; thus one element of **q***_ikj_* is 1 and the rest are zero. Note that **a***_kjs_* is an *n*_f_ × 1 vector of sizes of effects for each potential QTL and *u_pis_* is a polygenic effect.

If we place the effects for all environments or traits in a single vector (as a row vector) for line *i*,

$$u_{gi}^{\text{T}}=\sum_{k=1}^{c}\sum_{j=1}^{r_{k}-1}q_{ikj}^{T}A_{kj}+u_{pi}^{T},$$

where **A***_kj_* is a *n*_f_ × *t* matrix of sizes of effects for *n*_f_ founder alleles and *t* environments or traits and **u***_pi_* is a vector of polygenic effects for line *i* for all environments or traits. Placing the total genetic effects $u_{gi}^{\text{T}}$ for all lines as the rows in an *n*_g_ × *t* matrix **U**_g_, the model becomes

$$U_{\text{g}}=\sum_{k=1}^{c}\sum_{j=1}^{r_{k}-1}Q_{kj}A_{kj}+U_{\text{p}},$$ (3)

where **Q***_kj_* is an *n*_g_ × *n*_f_ matrix with *i*th row $q_{ikj}^{T}.$

Each vector **q***_ikj_* has a multinomial distribution with sample size 1 and a vector consisting of the probability of inheriting each founder allele for line *i* in interval *j* on linkage group *k*; we denote the vector of probabilities by **p***_ikj_*. Then

$$E\left(q_{ikj}\right)=p_{ikj}.$$

The regression approach for QTL mapping is used (Haley and Knott 1992) and so (3) is replaced by

$$U_{\text{g}}=\sum_{k=1}^{c}\sum_{j=1}^{r_{k}-1}P_{kj}A_{kj}+U_{\text{p}},$$

where **P***_kj_* is the matrix of probabilities with *i*th row $p_{ikj}^{T}.$ If we form the vector of genetic line effects by stacking the columns of **U**_g_, we find

$$u_{\text{g}}=\left(I_{t}⊗P\right)a+u_{\text{p}},$$ (4)

where if $r=\sum_{k=1}^{c}r_{k},$ **P** is a *n*_g_ × (*r* − *c*)*n*_f_ matrix of probabilities and **a** is the vector of potential QTL sizes ordered as founders in intervals (or markers). Our working model is given by $a∼N\left(0,G_{a}⊗I_{\left(r-c\right)n_{\text{f}}}\right),$ where **G***_a_* specifies a model for the genetic variances for environments or traits and covariances between environments and traits for the sizes of potential QTL effects.

Determination of **P** is discussed in Verbyla *et al.* (2014) for single-environment or -trait analysis. The use of three-point probabilities and probabilities based on a hidden Markov model (HMM) (Broman 2006) was discussed and examined in that article. An averaging over each interval was used to determine probabilities for interval-based analysis. It was found in a simulation study that the probabilities found using HMM led to reduced false positives while maintaining power of detection of QTL. HMM-based probabilities were used in the extension to multivariate situations discussed in this article.

The actual selection of putative QTL proceeds by forward selection, one QTL at a time. This requires a suitable test at each stage of potential selection.

### Threshold for QTL selection

A multienvironment or multitrait QTL exists if **G***_a_* ≠ **0**. Thus we test the hypothesis H_0_: **G***_a_* = **0** to establish whether a QTL exists. If the test is rejected, there is evidence that at least one putative QTL exists and a process (described below) is used to select the most likely interval for the putative QTL. If the test is retained, the selection process concludes.

The test of H_0_: **G***_a_* = **0** is nonstandard and is discussed in Verbyla and Cullis (2012). The process involves fitting two models, which have diagonal matrices for the genetic effects, potential QTL, and polygenic effects. The test then is equivalent to H_0_: tr(**G***_a_*) = 0. Thus if $\hat{\ell }$ is the maximized residual log-likelihood including the diagonal variance model for putative QTL sizes and the polygenic effects, and ${\hat{\ell }}_{0}$ is the maximized residual log-likelihood omitting the diagonal variance model for the QTL effects, the likelihood-ratio test statistic is found by

$$X_{\text{LR}}^{2}=2\left(\hat{\ell }-{\hat{\ell }}_{0}\right).$$ (5)

The statistic (5) has an approximate distribution under the null hypothesis (zero diagonal variance matrix) that is a mixture of chi-square distributions (Stram and Lee 1994); namely

$$X_{\text{LR}}^{2}∼{\left(\frac{1}{2}\right)}^{t}\sum_{k=0}^{t}\left(\begin{array}{c} t\\ k \end{array}\right)\chi_{k}^{2},$$ (6)

where $\chi_{k}^{2}$ represents a chi-square distribution on *k* d.f. Thus a test of size *α* of the hypothesis that the diagonal **G***_a_* is zero is rejected if $X_{\text{LR}}^{2}>c_{1-\alpha },$ where the critical value *c*_1−_*_α_* is determined using (6). This establishes the presence of variation that is necessary for a QTL to exist.

### Outlier statistic for QTL selection

If the test of H_0_: **G***_a_* = **0** is rejected, a model with correlated genetic effects (both putative QTL and polygenic) across traits or environments is fitted and based on that fitted model, an outlier statistic is used to select the putative QTL.

The process for selecting a QTL in the biparental situation is presented by Verbyla and Cullis (2012). The same argument leads to the statistic

$$t_{kj}^{2}=\frac{\sum_{f=1}^{n_{\text{f}}}{\tilde{a}}_{kjf}^{\text{T}}G_{a}^{-}{\tilde{a}}_{kjf}}{\sum_{f=1}^{n_{\text{f}}}\text{tr}\left(G_{a}^{-}\mathrm{var}\left({\tilde{a}}_{klf}\right)\right)}$$ (7)

so that for MAGIC it is necessary to sum over the founders; thus ${\tilde{a}}_{kjf}$ is the best linear unbiased predictor of the vector of sizes for all the environments or traits for the *j*th interval on linkage group *k* for founder *f* and $G_{a}^{-}$ is the generalized inverse of **G***_a_*. The interval (or marker) with the largest (outlier) statistic (7) is selected as the putative QTL and is added to the model as a random effect(s).

### Revised models

If a putative QTL is selected, the models (2) and (4) are revised by adding the QTL to the models as a random effect(s). The procedure above is then repeated to examine whether a further QTL can be added, until the test of H_0_: **G***_a_* = **0** is not rejected.

The revised models depend on whether the analysis is multienvironment or multitrait in nature. The reason for the difference is because for multienvironment analysis it is sensible to examine whether the QTL has the same expression at all sites or there is QTL-by-environment interaction. Thus the multienvironment model contains a main effect that is common across environments and environment-specific effects that allow for departures from the common effect. These latter effects can be tested for significance to establish whether the putative QTL sizes are common across environments or are different across environments.

Let **a**_−1_ be the vector of possible sizes omitting the trait- or environment-by-founder effects for the first QTL; the corresponding matrix of remaining probabilities is given by **P**_−1_. If **P**_1_ is the *n*_g_ × *n*_f_ matrix of probabilities corresponding to the first QTL chosen, for a multitrait analysis the model (2) becomes

$$u_{\text{g}}=\left(I_{t}⊗P_{1}\right)a_{1t}+u_{\text{p}}$$

and (4) becomes

$$u_{\text{g}}=\left(I_{t}⊗P_{1}\right)a_{1t}+\left(I_{t}⊗P_{-1}\right)a_{-1}+u_{\text{p}},$$

where **a**_1_*_t_* is the vector of trait-by-founder effect sizes for the first QTL. Note that it is assumed that $a_{1t}∼N\left(0,\text{diag}\left(\sigma_{1s}^{2}\right)⊗I_{n_{\text{f}}}\right),$ so that the putative QTL effects are modeled as random effects with their own variance matrix with each trait having its own variance.

For a multienvironment analysis (2) is updated to

$$u_{\text{g}}=\left(1_{t}⊗P_{1}\right)a_{1}+\left(I_{t}⊗P_{1}\right)a_{1t}+u_{\text{p}}$$

and (4) is updated to

$$u_{\text{g}}=\left(1_{t}⊗P_{1}\right)a_{1}+\left(I_{t}⊗P_{1}\right)a_{1t}+\left(I_{t}⊗P_{-1}\right)a_{-1}+u_{\text{p}},$$

where $a_{1}∼N\left(0,\sigma_{1}^{2}I_{n_{\text{f}}}\right)$ is the vector of sizes for each founder for the QTL chosen; this is the same for all environments and hence the **1***_t_* in the design matrix for the term containing **a**_1_. For the multienvironment situation, it is also assumed $a_{1t}∼N\left(0,\sigma_{1t}^{2}I_{t}⊗I_{n_{\text{f}}}\right),$ which differs from the multitrait situation. The two components allow for a simple QTL-by-environment model in which the common effect allows for correlation between environments, much in the same manner as the simplest variance component mode for genotype-by-environment modeling.

The process is now repeated until the test for possible QTL is not rejected. If the number of putative QTL selected is *l*, **P***_j_* is the vector of probabilities for all lines that correspond to QTL *j*, and **P***_−l_* is the matrix of probabilities omitting all **P***_j_*, the final model for a multitrait analysis is given by

$$u_{\text{g}}=\sum_{j=1}^{l}\left(I_{t}⊗P_{j}\right)a_{jt}+\left(I_{t}⊗P_{-l}\right)a_{-l}+u_{\text{p}},$$

where $a_{jt}∼N\left(0,\text{diag}\left(\sigma_{js}^{2}\right)⊗I_{n_{\text{f}}}\right)$ or for a multienvironment analysis

$$u_{\text{g}}=\sum_{j=1}^{l}\left\{\left(1_{t}⊗P_{j}\right)a_{j}+\left(I_{t}⊗P_{j}\right)a_{jt}\right\}+\left(I_{t}⊗P_{-l}\right)a_{-l}+u_{\text{p}},$$ (8)

where $a_{j}∼N\left(0,\sigma_{j}^{2}I_{n_{\text{f}}}\right)$ and $a_{jt}∼N\left(0,\sigma_{jt}^{2}I_{t}⊗I_{n_{\text{f}}}\right).$

The multitrait model specifies a diagonal form for the trait-by-QTL combinations. This is a very simplistic model. It would be preferable to include correlations between traits. The main reason correlations have not been included is that they cannot be fitted due to limited information; the variances and correlations in essence depend on the vector of sizes. All attempts to fit models with correlation in simulations or real studies failed. In addition, if an estimated variance for a trait or environment is zero (or very close to zero), correlations between that trait and other traits are not defined. This makes fitting of models very difficult. Thus while the diagonal matrix is not ideal, it represents a solution that seems to work in practice.

In the multienvironment situation, the environments are correlated but in a very simple way. It would be preferable to model both QTL variances and correlations more generally, but the same computational difficulties exist as for multitrait analyses.

### Test of QTL-by-environment interaction

For the multienvironment situation, it is of interest to test for QTL-by-environment interaction. Thus we wish to test ${\text{H}}_{0}\;:\sigma_{jt}^{2}=0,$ which is nonstandard, just like the test for possible selection of a QTL. The same form of test is used to examine this interaction for every QTL, using a residual log-likelihood-ratio statistic for the final model (8) and a null hypothesis model with the appropriate term in **a***_jt_* removed. The null distribution is a mixture of a point probability of 0.5 at zero and one-half a chi-square distribution with 1 d.f. (Stram and Lee 1994). Each QTL is examined in turn, allowing for all other effects, and model reduction is carried out according to these tests.

### Significance, LOGP scores, and percentage of variance

The significance, calculation of a measure of strength of a putative QTL, and percentage of variance of each QTL are all assessed in a manner similar to that presented in Verbyla *et al.* (2014), for each trait or environment.

We begin with a measure of significance. If the analysis is multitrait, the vector of QTL sizes for trait *s* is $a_{js}^{*}=a_{js}$ while in the multienvironment case it is $a_{js}^{*}=a_{j}+a_{js}$ if QTL-by-environment interaction is present or $a_{js}^{*}=a_{j}$ if QTL-by-environment interaction is not present. Then under the normality assumptions for a linear mixed model,

$$a_{js}^{*}|y_{2}∼N\left({\tilde{a}}_{js}^{*},V_{js}\right),$$

where **y**_2_ is the component of the data free of fixed effects (Verbyla 1990). The mean of this conditional distribution is the best linear unbiased prediction of $a_{js}^{*},$ that is, the estimated size of the QTL ${\tilde{a}}_{js}^{*},$ and **V***_js_* is the prediction error variance matrix (PEV) of $a_{js}^{*}.$ If $V_{js}^{-}$ is a generalized inverse of **V***_js_*, the distance measure

$$d_{js}^{2}={\left(a_{js}^{*}-{\tilde{a}}_{js}^{*}\right)}^{\text{T}}V_{js}^{-}\left(a_{js}^{*}-{\tilde{a}}_{js}^{*}\right)$$

has a chi-square distribution on *n*_f_ − 1 d.f. If

$$c_{js}^{2}={\tilde{a}}_{js}^{*\text{T}}V_{js}^{-}{\tilde{a}}_{js}^{*},$$

a measure of the strength of the putative QTL is given by

$$p_{js}=\text{Pr}\left(d_{js}^{2}>c_{js}^{2}\right).$$

This probability can be calculated for the QTL as a whole, that is, for all founders together, and also for individual founders, enabling “significance” of QTL effects both at the overall and at the founder level to be reported.

To measure the strength of a putative QTL, the probability *p_js_* is transformed using

$${\text{LOGP}}_{js}=-\log_{10}\left(p_{js}\right)$$

and this measure, LOGP, is similar to a LOD score.

Finally, the (approximate) percentage of genetic variance explained by each QTL can be found as follows. Consider the genetic effect for line *i* for trait or environment *s* in terms of the indicator variable **q***_ij_* for QTL *j* for line *i*,

$$u_{gis}=\sum_{j=1}^{l}q_{ij}^{\text{T}}a_{js}^{*}+p_{i,-l}^{\text{T}}a_{-l}+u_{pis},$$

where $p_{i,-l}^{\text{T}}$ is the *i*th row of **P**_−_*_l_*. Then the variance of *u_gis_* is approximately given by

$$\text{var}\left(u_{gis}\right)=\sum_{j=1}^{l}a_{sj}^{*\text{T}}\text{var}\left(q_{ij}\right)a_{js}^{*}+\sigma_{a}^{2}p_{i,-l}^{\text{T}}p_{i,-l}+\sigma_{ps}^{2}.$$

To evaluate var(**q***_ij_*), we proceed as in Verbyla *et al.* (2014) and define an “average” line and hence an average QTL indicator ${\bar{q}}_{j}$ so that an overall approximate variance can be found. The average founder probabilities, ${\bar{p}}_{j}$, found by averaging those probabilities over the lines are used and the multinomial nature of ${\bar{q}}_{j}$ means that

$$\text{var}\left({\bar{q}}_{j}\right)=\text{diag}\left({\bar{p}}_{j}\right)-{\bar{p}}_{j}{\bar{p}}_{j}^{\text{T}}.$$

For the term involving the intervals (or markers) not selected, we simply average the founder probabilities over all the lines for the nonselected intervals (or markers), ${\bar{p}}_{-l}$ say. Then the total variance of an average line effect, $u_{gs}^{*}$ is defined as

$$\text{var}\left(u_{gs}^{*}\right)=\sum_{j=1}^{l}a_{js}^{*\text{T}}\text{var}\left({\bar{q}}_{j}\right)a_{js}^{*}+\sigma_{as}^{2}{\bar{p}}_{-l}^{\text{T}}{\bar{p}}_{-l}+\sigma_{ps}^{2}.$$

The percentage of genetic variance attributed to the *j*th QTL is then

$${\text{PV}}_{js}=100\frac{a_{js}^{*\text{T}}\text{var}\left({\bar{q}}_{j}\right)a_{js}^{*}}{\text{var}\left(u_{\text{g}}^{*}\right)}.$$

In practice the unknown sizes $a_{js}^{*}$ and variance components $\sigma_{as}^{2}$ and $\sigma_{ps}^{2}$ are replaced by their estimates.

### Dimension reduction

The dimension reduction discussed by Verbyla *et al.* (2012) and used in univariate MAGIC QTL analysis (Verbyla *et al.* 2014) can be utilized in the multivariate situations discussed in this article. Thus a model that is equivalent to (4) is

$$u_{\text{g}}=\left(I_{t}⊗{\left(PP^{\text{T}}\right)}^{1/2}\right)a^{*}+u_{\text{p}},$$ (9)

where **a**∗ is an *n*_g_ × 1 vector with assumed distribution $N\left(0,G_{a}⊗I_{n_{\text{g}}}\right).$ The model (9) then generates the same variance model as (4) and the predicted random effects for the original model can be recovered from those found using (9) as

$$\tilde{a}=\left(I_{t}⊗P^{\text{T}}{\left(PP^{\text{T}}\right)}^{-1/2}\right){\tilde{a}}^{*}$$

with variance matrix

$$\text{var}\left(\tilde{a}\right)=(I_{t}⊗P^{\text{T}}{\left(PP^{\text{T}}\right)}^{-1/2}\text{var}\left({\tilde{a}}^{*}\right){\left(I_{t}⊗PP^{\text{T}}\right)}^{-1/2}P$$

and only diagonal elements of this matrix are required in computing the outlier statistics (7). Thus the computations for model fitting have a dimension of the number of lines rather than the number of intervals or markers.

### Computation

The computations were carried out in R (R Development Core Team 2013), using packages asreml (Butler *et al.* 2011) and components of wgaim (Taylor and Verbyla 2011). The required functions, including a likelihood-ratio routine for testing QTL-by-environment interaction and summary methods for displaying results of an analysis, are in the mpwgaim package in R available from the authors. Note also that a worked example is available in Supporting Information, File S1.

## Materials

### Simulation study

#### Genetic data for simulation studies:

The genetic data were generated for the simulations, using the mpMap package (Huang and George 2011) in R (R Development Core Team 2013).

A classic four-way population of 500 individuals was simulated, so that there were two crosses between two pairs of founder lines (F_1_ × F_2_ and F_3_ × F_4_). A line from each cross was crossed and then 500 lines were generated from that cross. These lines were selfed for six generations.

The linkage map was simulated to have seven chromosomes, each of length 300 cM and each with 201 markers equally spaced (1.5-cM spacing). The marker data were simulated for both founders and the 500 selfed lines, using mpMap.

#### Null simulation study:

Two hundred simulations were conducted for the case where no QTL were present, to assess the type I error rate and the average number of QTL detected per simulation. Phenotypic data for each simulation were generated for the simulated 500 MAGIC lines with two replicates and three traits in a MAGIC population, using the model (*i* = 1, 2, … , 500; *j* = 1, 2, 3; *k* = 1, 2)

$$y_{ijk}=\mu_{j}+u_{pij}+e_{ijk},$$

where *μ_j_* were 9, 10, and 12; the errors *e_ijk_* were independent standard normal; and the polygenic effects *u_pij_* were simulated having zero mean and covariance matrix

$$G_{\text{p}}=\frac{1}{2}\left[\begin{array}{ccc} 1.0 & 0.7 & 0.5\\ 0.7 & 1.0 & 0.5\\ 0.5 & 0.5 & 1.0 \end{array}\right]$$ (10)

for the three environments for each line.

#### Power simulation study:

A simple simulation to examine the power of the MVMPWGAIM approach was conducted. The 500 simulated MAGIC lines (with two replicates) and the linkage map used in the null simulation study were also used in the power study. Data were again simulated for three traits, now with four QTL. The generating model for the phenotype was (*i* = 1, 2, … , 500; *j* = 1, 2, 3; *k* = 1, 2)

$$y_{ijk}=\mu_{j}+\sum_{l=1}^{4}q_{il}^{\text{T}}a_{jl}+u_{pij}+e_{ijk},$$ (11)

where the means *μ_j_* were as for the null simulation, the polygenic effects *u_pij_* were defined as for the null distribution simulation, including (10), and *e_ijk_* were assumed independent and identically distributed as standard normal variables.

The vector **q***_il_* in (11) is the vector of founder indicators and for each line *i*, one element is one and the rest are zero. The element that is 1 indicates which founder provides the QTL allele. The vector of QTL sizes is **a***_jl_*, of length 4 (one for each founder), and there are 3 × 4 such vectors of sizes corresponding to the three traits and four QTL. The sizes of QTL effects used in the simulations are given in Table 1. The percentage of genetic variance explained by each QTL for each trait was calculated using the given sizes and the multinomial nature of those sizes; the calculation mirrored the development presented in the *Methods* section. It was assumed that all founder alleles were equally likely to be inherited, that is, 0.25 for the four founders.

**Table 1:** Specification for the power simulation

		Environment 1					Environment 2					Environment 3				
		Founder					Founder					Founder				
Chr	Position (cM)	1	2	3	4	% var	1	2	3	4	% var	1	2	3	4	% var
1	141	0.3	−0.3	−0.3	0.3	11.7	0.3	−0.3	−0.3	0.3	11.7	0.3	−0.3	−0.3	0.3	11.7
2	160	0.3	−0.3	−0.3	0.3	11.7	0.3	−0.3	−0.3	0.3	11.7	−0.3	0.3	0.3	−0.3	11.7
3	174	0.0	0.0	0.0	0.0	0.0	0.0	0.0	0.0	0.0	0.0	0.3	−0.3	−0.3	0.3	11.7
4	207	0.3	−0.3	−0.3	0.3	11.7	0.3	−0.3	−0.3	0.3	11.7	0.0	0.0	0.0	0.0	0.0
Total						35.1					35.1					35.1

Four QTL on chromosomes (Chr) 1–4 are shown with sizes as specified for the founder alleles for each of three environments together with percentage of genetic variance (% var) explained by each QTL at each environment.

The simulation is an attempt to examine some simple scenarios to see how successful is the detection of QTL using MVMPWGAIM. The first QTL has the same pattern of sizes across all traits, a pleiotropic QTL. The second QTL has the same vector of sizes for traits 1 and 3 but opposite signs for trait 2. The third QTL is absent for traits 1 and 2. Finally, trait 2 does not express for the fourth QTL.

In the power study, a QTL was declared as detected if the interval selected was within 5 cM either side of the true QTL position. For the scenario presented, 200 simulations were carried out.

### Examples

#### Linkage map:

A linkage map for a four-way cross in wheat developed in the Commonwealth Scientific and Industrial Research Organisation (CSIRO), Australia, was described in Verbyla *et al.* (2014). There were 5763 markers on the map, and most were SNPs with 755 DArT markers (Diversity Arrays Technology) and 39 multiallelic microsatellites, grouped into 21 chromosomes of wheat together with three additional linkage groups. The total map length was 5788 cM.

Many of the markers were colocated at the same position on the map and so markers were removed prior to QTL analysis to ensure nonzero recombination fractions between the remaining markers. The final map for QTL analysis consisted of 3230 markers (including 620 DArTs and 37 microsatellites).

The following files are related to the genetic data: The linkage map is presented in File S2; the pedigree information for the MAGIC RIL lines and the founders is given in File S3, while the correspondence between the pedigree identifiers and the identifiers in the phenotypic data is given in File S4; and the marker data for the founders and RILs are given in File S5 and File S6, respectively.

#### Seed size in wheat:

Two important measures of seed size in wheat are hectoliter weight (HW) and the weight of 1000 kernels (TKW) due to their impact on milling quality and yield. These two variables were measured on the four-way wheat MAGIC population developed by CSIRO in Australia in a field trial at Yanco, New South Wales in 2009. The trial was designed as a partially replicated experiment with 53% of 1063 four-way lines replicated and the rest unreplicated. The layout was 81 rows by 20 columns, in three blocks of 27 rows. The data are available in File S7.

Individual trait analyses were conducted for the two traits, using the methods of Gilmour *et al.* (1997). The blocking factor in the design was included in each model. The two traits exhibited some spatial variation, similar for both traits, that was included in the model. The only other extraneous or global effect that was found in the data was a random effect for rows that was required for the 1000-kernel weight analysis. The heritability for hectoliter weight was 77% and for 1000-kernel weight it was 85%.

The bivariate models that are required for the multitrait QTL analysis were of the (symbolic) form

$$\begin{array}{l} y=Trait.Type+Trait.Block+at\left(Trait,\;2\right).Row\\ +\;Trait.Genotype+error, \end{array}$$

where *y* is the response variable, composed of HW and TKW arranged suitably in a vector, *Trait* denotes the factor for the two traits that indicates the corresponding trait for each value in *y*, *Genotype* is the indicator for the line for each *y*, *Block* denotes the blocking factor, and *error* is the residual error variable. The factor *Type* separates out the four-way lines of interest from other lines that were planted in the trial; these other lines consisted of the four-way founders and other standard commercial wheat varieties. The replication of these additional lines varied but in general these lines had several replicates. The meaning of “.” depends on what other terms are in the model, but they generally are interactions or simply combinations of variables. In the symbolic model above, the *Trait.Genotype* term is nested in *Trait.Type*.

In the model, the term *Trait.Type* is a fixed-effects term, that is, corresponds to **Xτ** in (1), that allows a different mean for each trait and type combination. The other effects were taken as random. The term *Trait.Block*, which allows for a different block effect for each trait, and the term *at*(*Trait*, *2*).*Row*, which allows for a random row effect for the second trait, here 1000-kernel weight, are components of **Z**_o_**u**_o_ in (1).

*Trait.Genotype* allows for variation of genotypes for each trait [**Z**_g_**u**_g_ in (1)], and the form of this variation depends on the structure imposed on the *Trait* factor. For example, effects associated with this factor might be random with zero mean vector (2 × 1) and a diagonal variance matrix with separate variance for each trait. Alternatively, we would in general allow for a correlation between the traits; this is the genetic correlation between traits. These model are also appropriate for *Trait.Block*.

The *error* term also has a model, namely a separable structure that corresponds to a three-way combination of factors, namely *Trait.Column.Row*. The *Trait* component is treated in the same manner as the terms preceding it, while we impose an autoregressive process of order 1 for both the column and the row components. This generates a three-way separable variance matrix.

The bivariate models required for QTL analysis have first diagonal structures for the *Trait* component in each term to establish whether a putative QTL should be selected and, for QTL selection, a 2 × 2 variance matrix that allows for different variances for the two traits and a correlation between traits.

The analysis with correlation between the traits resulted in a genetic correlation of 0.28 and genetic variances of 2.6 and 14.1 for HW and TKW, respectively. The genetic correlation is not large but is present so we expect some pleiotropic QTL. The correlation between the traits at the residual level was 0.26 and residual variances were 1.1 and 3.2 for HW and TKW, respectively. The spatial correlation in the column direction was close to zero while it was 0.16 in the row direction.

#### Multienvironment trials for flowering time:

Three trials were conducted using the MAGIC four-way wheat population of CSIRO, Australia. One of the aims was to investigate flowering time, using zadok scores (Zadok *et al.* 1974). Plant maturity or flowering time is an important adaptation trait (Worland 1996) that breeders either directly or indirectly select for in breeding. The development from the vegetative phase to the reproductive phase can be divided into three components, vernalization requirement (VR), photoperiod sensitivity (PS), and earliness *per se* (eps). Each stage is controlled by different genes and influences the overall flowering time.

The trials were conducted in Yanco, in 2009 (the same trial as the seed size experiment above), and at Leeton and Temora in 2010; all sites are in New South Wales. The numbers of four-way lines used in the trials were 1063, 1026, and 1025 for Yanco, Leeton, and Temora, respectively. The trials were all partially replicated designs (53%, 44%, and 56% replication, respectively). The layouts as rows by columns were 81 × 20, 40 × 46, and 90 × 16, respectively. The blocking structure varied across trials: Yanco had three blocks of 27 rows; Leeton had two blocks of 20 rows; and Temora was blocked in two directions, three blocks of 30 rows and two blocks of 8 columns. The data are available in File S8.

The analyses for the individual trials resulted in models that included the blocking structures in the experimental design for each trial and a separable autoregressive of order 1 for rows and columns (Gilmour *et al.* 1997) at the error level, thereby modeling the spatial correlation. No other terms were necessary. The heritabilities for zadok score for the three trials were 82%, 82%, and 92%, respectively, which are very high.

The multienvironment model was of the (symbolic) form

$$\begin{array}{l} zadok=Site.Type+Site.Block+at\left(Site,\;2\right).Cblock\\ +\;Site.Genotype+error. \end{array}$$

The terms are similar to those explained for the seed size analysis. *Site* is the factor of three levels for the trials, *Cblock* is the blocking in the column direction for Temora [hence the term *at*(*Site*, *2*)], and *Site.Type* is a fixed effect to ensure correct mean effects are obtained for four-way and non-four-way lines. The other terms in the model are random effects. The *Site* variable is again modeled using a diagonal variance matrix or a 3 × 3 variance matrix that includes correlations between the sites. The *error* represents *at*(*Site*)*.Column.Row*, which allows for separate spatial models for each site, consisting of separable autoregressive processes of order 1 for rows and columns. The two models required for QTL analysis provide for the *Site* variance matrix to be diagonal or a general 3 × 3 structure for the *Site.Genotype* term.

The estimated genotype-by-environment (*Site.Genotype*) covariance/correlation matrix for the data was (correlations above the diagonal, variances down the diagonal, and covariances below the diagonal)

$$\begin{array}{l} \;\;\;\begin{array}{ccc} \text{Leeton} & \text{Temora} & \text{Yanco} \end{array}\\ \begin{array}{c} \text{Leeton}\\ \text{Temora}\\ \text{Yanco} \end{array}\left[\begin{array}{ccc} \;24.4 & \;0.92 & \;0.93\\ \;20.0 & \;19.7 & \;0.88\\ \;25.3 & \;21.5 & \;30.1 \end{array}\right] \end{array}$$ (12)

so that the three environments are highly genetically correlated.

## Results

### Simulation study

#### Null simulation:

For the null QTL simulation, the type I error rate was 0.035 with an average number of false positive QTL per simulation of 0.045. Thus the testing procedure is conservative when compared to the nominal level of 0.05 that was used.

#### Power simulation:

The rate of detection of each QTL (and the overall total across QTL) is presented in Table 2. On average three of four of the QTL were detected. This is probably due in part to the small size of the simulated MAGIC population, but also because the QTL that express in a subset of the traits (QTL on chromosomes 3 and 4) are more difficult to detect. This is similar to the results found in Verbyla and Cullis (2012). Perhaps more surprising is the lower rate of detection of the QTL on chromosome 1 compared to that on chromosome 2, although the rates for both are quite high.

**Table 2:** Proportion of correct determination (within 5 cM of the true QTL position) in 200 simulations for each of the four QTL, together with a total out of four

	QTL				
	1	2	3	4	Total
Proportion	0.880	0.990	0.415	0.720	3.005

The number of false positives was very low and is given in Table 3. The proportion per chromosome per simulation is ∼0.02. Overall the proportion per simulation was 0.125.

**Table 3:** Proportion of false positives over 200 simulations for each QTL chromosome and chromosomes 5–7 that contained no QTL

	Chromosome					
	1	2	3	4	5–7	Total
Proportion	0.020	0.020	0.030	0.010	0.045	0.125

The estimated mean QTL sizes and the standard error of the mean are given in Table 4. In general the sizes tend to be underestimated and it is conjectured that this might in part be due to the small size of the simulated MAGIC population. Verbyla and Cullis (2012) show this type of bias reduces for multivariate QTL analysis in biparental populations as the population size increases.

**Table 4:** Means and standard errors of the mean for each QTL and every founder size for the three traits in the 200 simulations

			Founder							
			1		2		3		4	
QTL	Trait	Method	Mean	SE	Mean	SE	Mean	SE	Mean	SE
1	Trait 1	Simulated	0.300	0.006	−0.300	0.006	−0.300	0.006	0.300	0.006
		Estimated	0.253		−0.266		−0.256		0.268	
	Trait 2	Simulated	0.300	0.006	−0.300	0.007	−0.300	0.006	0.300	0.006
		Estimated	0.257		−0.261		−0.255		0.258	
	Trait 3	Simulated	0.300	0.006	−0.300	0.006	−0.300	0.006	0.300	0.005
		Estimated	0.265		−0.266		−0.284		0.284	
2	Trait 1	Simulated	0.300	0.006	−0.300	0.006	−0.300	0.006	0.300	0.006
		Estimated	0.266		−0.293		−0.249		0.276	
	Trait 2	Simulated	0.300	0.005	−0.300	0.005	−0.300	0.005	0.300	0.006
		Estimated	0.271		−0.295		−0.259		0.283	
	Trait 3	Simulated	−0.300	0.005	0.300	0.005	0.300	0.006	−0.300	0.006
		Estimated	−0.277		0.292		0.283		−0.298	
3	Trait 1	Simulated	0.000	0.004	0.000	0.004	0.000	0.04	0.000	0.05
		Estimated	0.000		−0.004		0.002		0.001	
	Trait 2	Simulated	0.000	0.003	0.000	0.002	0.000	0.003	0.000	0.003
		Estimated	0.000		−0.003		0.001		0.001	
	Trait 3	Simulated	0.300	0.008	−0.300	0.009	−0.300	0.008	0.300	0.008
		Estimated	0.295		−0.304		−0.292		0.302	
4	Trait 1	Simulated	0.300	0.006	−0.300	0.006	−0.300	0.007	0.300	0.007
		Estimated	0.265		−0.287		−0.265		0.287	
	Trait 2	Simulated	0.300	0.006	−0.300	0.007	−0.300	0.007	0.300	0.007
		Estimated	0.255		−0.289		−0.260		0.294	
	Trait 3	Simulated	0.000	0.003	0.000	0.003	0.000	0.004	0.000	0.003
		Estimated	−0.003		0.005		0.001		−0.003	

### Examples

#### Seed size:

The putative QTL found in the bivariate analysis are given in Table 5. There were 29 putative QTL found in the analysis. In total, the QTL explain 48% and 56% of the genetic variance for HW and TKW, respectively. The major contribution to HW comes from a QTL on 7B, while that for TKW comes from 2D.

**Table 5:** Results for bivariate QTL analysis (using intervals) of hectoliter weight, labeled HW, and 1000-kernel weight, labeled TKW

Chromosome	Dist (cM)	Dist (cM)	Trait	Founder	Size	Founder prob	Founder LOGP	Prob	% var	LOGP
1A	37.16	40.25	HW	Yitpi	−0.174	0.2265	0.65	0.0002	3.5	3.6
				Chara	−0.474	0.0236	1.63			
				Baxter	0.402	0.0465	1.33			
				Westonia	0.194	0.2072	0.68			
			TKW	Yitpi	0.000	0.4997	0.30	1.0000	0.0	0.0
				Chara	0.000	0.4997	0.30			
				Baxter	0.000	0.4992	0.30			
				Westonia	0.000	0.4996	0.30			
1B	124.79	126.83	HW	Yitpi	−0.495	0.0113	1.95	0.0010	2.6	3.0
				Chara	0.188	0.1939	0.71			
				Baxter	0.033	0.4415	0.36			
				Westonia	0.233	0.1412	0.85			
			TKW	Yitpi	0.000	0.4999	0.30	1.0000	0.0	0.0
				Chara	0.000	0.4997	0.30			
				Baxter	0.000	0.4997	0.30			
				Westonia	0.000	0.5000	0.30			
1D	114.44	114.95	HW	Yitpi	−0.139	0.1732	0.76	0.0418	0.8	1.4
				Chara	0.009	0.4773	0.32			
				Baxter	−0.155	0.1778	0.75			
				Westonia	0.268	0.0453	1.34			
			TKW	Yitpi	−0.281	0.1274	0.89	0.2617	0.2	0.6
				Chara	0.000	0.4997	0.30			
				Baxter	0.359	0.0992	1.00			
				Westonia	−0.124	0.3199	0.49			
2A	220.64	221.65	HW	Yitpi	−0.017	0.3713	0.43	0.9554	0.0	0.0
				Chara	0.015	0.3893	0.41			
				Baxter	0.013	0.4010	0.40			
				Westonia	−0.012	0.4076	0.39			
			TKW	Yitpi	0.305	0.1475	0.83	0.0942	0.6	1.0
				Chara	0.205	0.2969	0.53			
				Baxter	−0.441	0.0915	1.04			
				Westonia	−0.142	0.3415	0.47			
2A	284.61	285.12	HW	Yitpi	0.283	0.0338	1.47	0.0481	0.8	1.3
				Chara	−0.033	0.4131	0.38			
				Baxter	−0.125	0.2066	0.68			
				Westonia	−0.142	0.1717	0.77			
			TKW	Yitpi	−0.239	0.1765	0.75	0.1803	0.3	0.7
				Chara	−0.084	0.3697	0.43			
				Baxter	−0.095	0.3535	0.45			
				Westonia	0.376	0.0655	1.18			
2B	0	0.5	HW	Yitpi	0.000	0.4995	0.30	1.0000	0.0	0.0
				Chara	0.000	0.4995	0.30			
				Baxter	0.000	0.4994	0.30			
				Westonia	0.000	0.4994	0.30			
			TKW	Yitpi	−0.499	0.1689	0.77	0.0224	0.8	1.6
				Chara	0.293	0.2371	0.63			
				Baxter	0.381	0.1720	0.76			
				Westonia	−0.306	0.2785	0.56			
2B	36.39	37.4	HW	Yitpi	−0.045	0.4315	0.36	0.0167	1.9	1.8
				Chara	0.296	0.0674	1.17			
				Baxter	−0.358	0.0748	1.13			
				Westonia	0.071	0.3941	0.40			
			TKW	Yitpi	0.000	0.4999	0.30	1.0000	0.0	0.0
				Chara	0.000	0.4997	0.30			
				Baxter	0.000	0.5000	0.30			
				Westonia	0.000	0.4998	0.30			
2B	81.9	82.4	HW	Yitpi	0.336	0.0617	1.21	0.0306	1.7	1.5
				Chara	−0.335	0.0881	1.05			
				Baxter	0.099	0.3526	0.45			
				Westonia	−0.138	0.2675	0.57			
			TKW	Yitpi	0.394	0.2546	0.59	0.0053	2.4	2.3
				Chara	−1.422	0.0153	1.81			
				Baxter	0.084	0.4512	0.35			
				Westonia	0.571	0.1707	0.77			
2B	117.41	118.42	HW	Yitpi	0.000	0.4994	0.30	1.0000	0.0	0.0
				Chara	0.000	0.4986	0.30			
				Baxter	0.000	0.4997	0.30			
				Westonia	0.000	0.4983	0.30			
			TKW	Yitpi	0.602	0.1801	0.74	0.0052	2.1	2.3
				Chara	−1.462	0.0123	1.91			
				Baxter	0.190	0.3950	0.40			
				Westonia	0.265	0.3537	0.45			
2B	140.39	141.91	HW	Yitpi	0.438	0.0893	1.05	0.0000	5.3	4.5
				Chara	−0.849	0.0133	1.88			
				Baxter	0.328	0.1629	0.79			
				Westonia	−0.037	0.4615	0.34			
			TKW	Yitpi	1.458	0.0397	1.40	0.0000	6.9	6.8
				Chara	−2.306	0.0113	1.95			
				Baxter	−0.232	0.3960	0.40			
				Westonia	0.085	0.4663	0.33			
2D	7.35	31.25	HW	Yitpi	0.000	0.4998	0.30	1.0000	0.0	0.0
				Chara	0.000	0.4997	0.30			
				Baxter	0.000	0.4998	0.30			
				Westonia	0.000	0.4997	0.30			
			TKW	Yitpi	−1.486	0.0752	1.12	0.0000	22.7	16.9
				Chara	0.537	0.3149	0.50			
				Baxter	−3.131	0.0018	2.75			
				Westonia	2.136	0.0230	1.64			
2D	90.57	92.09	HW	Yitpi	−0.015	0.4518	0.35	0.2369	0.4	0.6
				Chara	0.134	0.1313	0.88			
				Baxter	−0.168	0.0934	1.03			
				Westonia	0.040	0.3744	0.43			
			TKW	Yitpi	−0.505	0.1151	0.94	0.0001	1.9	3.9
				Chara	0.757	0.0308	1.51			
				Baxter	0.216	0.3062	0.51			
				Westonia	−0.649	0.0628	1.20			
2D	128.63	129.64	HW	Yitpi	−0.135	0.2189	0.66	0.0430	1.1	1.4
				Chara	0.288	0.0593	1.23			
				Baxter	−0.221	0.0990	1.00			
				Westonia	0.046	0.3973	0.40			
			TKW	Yitpi	−0.188	0.3646	0.44	0.0000	3.9	7.0
				Chara	0.210	0.3556	0.45			
				Baxter	−1.317	0.0073	2.14			
				Westonia	0.942	0.0428	1.37			
3A	57.34	57.84	HW	Yitpi	0.262	0.0519	1.28	0.0449	0.9	1.4
				Chara	−0.045	0.3963	0.40			
				Baxter	0.000	0.4993	0.30			
				Westonia	−0.235	0.0775	1.11			
			TKW	Yitpi	0.534	0.0504	1.30	0.0358	0.6	1.4
				Chara	0.025	0.4716	0.33			
				Baxter	−0.193	0.2853	0.54			
				Westonia	−0.455	0.0874	1.06			
3A	291.17	292.69	HW	Yitpi	0.287	0.0348	1.46	0.0214	1.1	1.7
				Chara	−0.148	0.1795	0.75			
				Baxter	0.014	0.4658	0.33			
				Westonia	−0.172	0.1394	0.86			
			TKW	Yitpi	0.023	0.4518	0.35	0.5795	0.1	0.2
				Chara	−0.205	0.1439	0.84			
				Baxter	0.021	0.4567	0.34			
				Westonia	0.142	0.2278	0.64			
3B	61.63	65.8	HW	Yitpi	0.341	0.0592	1.23	0.0133	1.7	1.9
				Chara	−0.031	0.4539	0.34			
				Baxter	−0.335	0.0497	1.30			
				Westonia	−0.011	0.4829	0.32			
			TKW	Yitpi	0.000	0.4997	0.30	1.0000	0.0	0.0
				Chara	0.000	0.4998	0.30			
				Baxter	0.000	0.4997	0.30			
				Westonia	0.000	0.5000	0.30			
3D	39.21	39.71	HW	Yitpi	0.000	0.4985	0.30	1.0000	0.0	0.0
				Chara	0.000	0.4996	0.30			
				Baxter	0.000	0.4984	0.30			
				Westonia	0.000	0.4998	0.30			
			TKW	Yitpi	0.567	0.0488	1.31	0.0086	0.9	2.1
				Chara	−0.259	0.2451	0.61			
				Baxter	0.133	0.3555	0.45			
				Westonia	−0.554	0.0778	1.11			
5A	245.09	250.92	HW	Yitpi	−0.368	0.0536	1.27	0.0009	2.5	3.1
				Chara	−0.260	0.1221	0.91			
				Baxter	0.409	0.0433	1.36			
				Westonia	0.172	0.2205	0.66			
			TKW	Yitpi	−0.450	0.1076	0.97	0.0086	1.2	2.1
				Chara	−0.245	0.2456	0.61			
				Baxter	−0.099	0.3978	0.40			
				Westonia	0.680	0.0269	1.57			
5A	289.62	290.12	HW	Yitpi	−0.071	0.3068	0.51	0.2161	0.4	0.7
				Chara	−0.134	0.1949	0.71			
				Baxter	0.208	0.0698	1.16			
				Westonia	−0.015	0.4577	0.34			
			TKW	Yitpi	0.001	0.4976	0.30	0.8713	0.0	0.1
				Chara	0.012	0.4700	0.33			
				Baxter	−0.097	0.2599	0.59			
				Westonia	0.075	0.3119	0.51			
5A	321.45	324.54	HW	Yitpi	−0.215	0.0846	1.07	0.1294	0.8	0.9
				Chara	−0.048	0.3957	0.40			
				Baxter	0.024	0.4494	0.35			
				Westonia	0.222	0.1003	1.00			
			TKW	Yitpi	−0.598	0.1553	0.81	0.0000	4.5	4.6
				Chara	0.068	0.4584	0.34			
				Baxter	−1.203	0.0375	1.43			
				Westonia	1.276	0.0198	1.70			
5B	190.22	190.72	HW	Yitpi	0.120	0.2333	0.63	0.0627	0.9	1.2
				Chara	−0.278	0.0537	1.27			
				Baxter	0.126	0.2384	0.62			
				Westonia	0.013	0.4788	0.32			
			TKW	Yitpi	−0.047	0.3141	0.50	0.9542	0.0	0.0
				Chara	0.022	0.4095	0.39			
				Baxter	0.013	0.4480	0.35			
				Westonia	0.008	0.4702	0.33			
5D	35.87	64.86	HW	Yitpi	0.027	0.4327	0.36	0.4512	0.4	0.4
				Chara	0.047	0.3890	0.41			
				Baxter	−0.189	0.1067	0.97			
				Westonia	0.104	0.2607	0.58			
			TKW	Yitpi	−0.603	0.1074	0.97	0.0229	1.6	1.6
				Chara	−0.553	0.1449	0.84			
				Baxter	0.665	0.0767	1.12			
				Westonia	0.293	0.2789	0.55			
6A	85.68	86.18	HW	Yitpi	0.000	0.4994	0.30	0.0468	1.4	1.3
				Chara	−0.232	0.1557	0.81			
				Baxter	−0.122	0.2572	0.59			
				Westonia	0.328	0.0397	1.40			
			TKW	Yitpi	0.329	0.1300	0.89	0.1230	0.4	0.9
				Chara	0.049	0.4475	0.35			
				Baxter	−0.461	0.0646	1.19			
				Westonia	0.017	0.4777	0.32			
6B	162.33	163.85	HW	Yitpi	0.378	0.0365	1.44	0.0125	1.8	1.9
				Chara	−0.260	0.1448	0.84			
				Baxter	0.065	0.3825	0.42			
				Westonia	−0.219	0.1402	0.85			
			TKW	Yitpi	−0.427	0.0890	1.05	0.1303	0.5	0.9
				Chara	−0.205	0.2937	0.53			
				Baxter	0.240	0.2337	0.63			
				Westonia	0.317	0.1509	0.82			
7A	161.15	161.66	HW	Yitpi	−0.163	0.2031	0.69	0.0028	1.8	2.5
				Chara	0.074	0.3509	0.45			
				Baxter	0.352	0.0308	1.51			
				Westonia	−0.294	0.0683	1.17			
			TKW	Yitpi	0.144	0.3067	0.51	0.1241	0.4	0.9
				Chara	−0.013	0.4821	0.32			
				Baxter	−0.449	0.0495	1.31			
				Westonia	0.262	0.1806	0.74			
7A	286.39	287.4	HW	Yitpi	0.112	0.2275	0.64	0.0772	0.8	1.1
				Chara	−0.263	0.0360	1.44			
				Baxter	0.049	0.3719	0.43			
				Westonia	0.088	0.2819	0.55			
			TKW	Yitpi	−0.271	0.2726	0.56	0.0003	2.0	3.6
				Chara	−0.046	0.4590	0.34			
				Baxter	−0.826	0.0344	1.46			
				Westonia	0.923	0.0218	1.66			
7B	6.66	7.17	HW	Yitpi	0.192	0.3063	0.51	0.0002	14.2	3.8
				Chara	0.572	0.1002	1.00			
				Baxter	−1.220	0.0037	2.43			
				Westonia	0.249	0.2750	0.56			
			TKW	Yitpi	−0.396	0.1220	0.91	0.1701	0.6	0.8
				Chara	−0.303	0.2466	0.61			
				Baxter	0.129	0.3873	0.41			
				Westonia	0.464	0.1316	0.88			
7B	92.71	95.28	HW	Yitpi	0.079	0.3138	0.50	0.1288	0.8	0.9
				Chara	−0.063	0.3746	0.43			
				Baxter	−0.221	0.1064	0.97			
				Westonia	0.188	0.1286	0.89			
			TKW	Yitpi	0.484	0.0617	1.21	0.1293	0.5	0.9
				Chara	−0.172	0.3268	0.49			
				Baxter	−0.072	0.4166	0.38			
				Westonia	−0.315	0.1626	0.79			
Unlinked3	2.02	4.06	HW	Yitpi	−0.257	0.0669	1.17	0.1132	0.7	1.0
				Chara	−0.005	0.4896	0.31			
				Baxter	0.138	0.2129	0.67			
				Westonia	0.106	0.3148	0.50			
			TKW	Yitpi	−0.461	0.1014	0.99	0.0627	0.8	1.2
				Chara	−0.024	0.4782	0.32			
				Baxter	0.483	0.0934	1.03			
				Westonia	−0.100	0.4149	0.38			

Twenty-nine QTL were found. Dist, distribution; Prob, probability.

After the QTL analysis, the polygenic variances for HW and TKW were 1.70 and 8.22 with a genetic correlation of 0.25. Thus the QTL have resulted in a reduction in polygenic variance of 42% in both traits. The polygenic correlation remains much the same as before the QTL analysis.

Univariate analyses were also conducted (see Table S1 and Table S2) to compare results with the bivariate analyses. There were 18–20 QTL identified for TKW and HW (respectively), and of these, 7 and 8 for HW and TKW (respectively) were in common across univariate and bivariate analyses when considering the position as the same as if they were within 5 cM. However, the main difference was that the position identified on each of the chromosomes differed.

#### Multienvironment trial for flowering time:

The multienvironment QTL analysis resulted in 16 QTL being found, with 11 being significant as judged by the probability measure presented in the *Methods* section. The putative QTL are listed in Table 6. The percentage of genetic variance explained by the QTL overall in each environment was 82.1%, 81.6%, and 82.4% for Leeton, Temora, and Yanco, respectively. Six of the putative QTL expressed in the same way across all environments; that is, common sizes for each QTL were appropriate for all environments and this can be seen in Table 6. These putative QTL were on 2D (in the interval adjacent to the PPD-D1 gene also found in the analysis), which contributes ∼7–9% of the genetic variance across the environments, and also on 4B, 6B, 7A, and 7B.

**Table 6:** Results for multienvironment QTL analysis (using intervals) for Yanco, Temora, and Leeton

Chromosome	Dist (cM)	Dist (cM)	Trait	Founder	Size	Founder prob	Founder LOGP	Prob	% var	LOGP
2B	246.43	247.44	Leeton	Yitpi	−0.06	0.45	0.35	0	0.63	2.8
				Chara	−0.36	0.22	0.66			
				Baxter	0.40	0.23	0.63			
				Westonia	−0.04	0.47	0.33			
			Temora	Yitpi	0.25	0.28	0.55	0	0.72	2.8
				Chara	−0.10	0.41	0.39			
				Baxter	−0.00	0.50	0.30			
				Westonia	−0.10	0.43	0.37			
			Yanco	Yitpi	0.14	0.38	0.42	0	0.55	2.8
				Chara	−1.04	0.01	1.88			
				Baxter	0.61	0.14	0.87			
				Westonia	0.26	0.32	0.49			
2B	81.9	82.4	Leeton	Yitpi	−0.47	0.20	0.70	0.65	0.07	0.18
				Chara	−0.84	0.09	1.05			
				Baxter	0.16	0.40	0.40			
				Westonia	1.09	0.02	1.63			
			Temora	Yitpi	−0.47	0.20	0.70	0.87	0.03	0.06
				Chara	−0.84	0.09	1.05			
				Baxter	0.16	0.40	0.40			
				Westonia	1.09	0.02	1.63			
			Yanco	Yitpi	−0.47	0.20	0.70	0.02	0.34	1.64
				Chara	−0.84	0.09	1.05			
				Baxter	0.16	0.40	0.40			
				Westonia	1.09	0.02	1.63			
2D	5.83	7.35	Leeton	Yitpi	4.07	0.01	1.97	0	8.4	10.4
				Chara	−3.52	0.03	1.54			
				Baxter	−0.13	0.48	0.32			
				Westonia	−1.13	0.32	0.49			
			Temora	Yitpi	4.07	0.01	1.97	0	9.61	10.4
				Chara	−3.52	0.03	1.54			
				Baxter	−0.13	0.48	0.32			
				Westonia	−1.13	0.32	0.49			
			Yanco	Yitpi	4.07	0.01	1.97	0	7.29	10.4
				Chara	−3.52	0.03	1.54			
				Baxter	−0.13	0.48	0.32			
				Westonia	−1.13	0.32	0.49			
2D	7.35	31.25	Leeton	Yitpi	−10.93	0.00	6.29	0	69.19	43.1
				Chara	7.22	0.00	3.20			
				Baxter	−4.67	0.04	1.39			
				Westonia	4.82	0.03	1.56			
			Temora	Yitpi	−10.25	0.00	5.75	0	68.01	52.2
				Chara	6.54	0.00	2.82			
				Baxter	−4.18	0.06	1.24			
				Westonia	4.62	0.03	1.50			
			Yanco	Yitpi	−10.89	0.00	6.25	0	69.11	47
				Chara	7.91	0.00	3.68			
				Baxter	−6.00	0.01	1.90			
				Westonia	5.51	0.01	1.85			
4B	86.73	87.24	Leeton	Yitpi	−0.15	0.36	0.44	0	0.51	3.2
				Chara	0.38	0.20	0.70			
				Baxter	0.63	0.08	1.09			
				Westonia	−0.89	0.02	1.72			
			Temora	Yitpi	−0.15	0.36	0.44	0	0.58	3.2
				Chara	0.38	0.20	0.70			
				Baxter	0.63	0.08	1.09			
				Westonia	−0.89	0.02	1.72			
			Yanco	Yitpi	−0.15	0.36	0.44	0	0.44	3.2
				Chara	0.38	0.20	0.70			
				Baxter	0.63	0.08	1.09			
				Westonia	−0.89	0.02	1.72			
5B	240.97	241.47	Leeton	Yitpi	0.62	0.09	1.05	0.01	0.48	2.3
				Chara	−0.98	0.02	1.73			
				Baxter	0.22	0.33	0.48			
				Westonia	0.05	0.46	0.34			
			Temora	Yitpi	0.10	0.41	0.39	0.65	0.07	0.2
				Chara	−0.02	0.48	0.32			
				Baxter	0.31	0.25	0.59			
				Westonia	−0.32	0.25	0.60			
			Yanco	Yitpi	0.45	0.17	0.78	0	0.76	4
				Chara	−1.33	0.00	2.58			
				Baxter	0.03	0.48	0.32			
				Westonia	0.81	0.05	1.26			
5D	64.86	68.49	Leeton	Yitpi	−0.17	0.37	0.44	0.06	0.22	1.2
				Chara	−0.34	0.24	0.62			
				Baxter	0.71	0.06	1.20			
				Westonia	−0.24	0.31	0.51			
			Temora	Yitpi	−0.09	0.43	0.37	0	0.43	2.7
				Chara	−0.50	0.14	0.84			
				Baxter	0.91	0.02	1.66			
				Westonia	−0.33	0.24	0.62			
			Yanco	Yitpi	−0.34	0.24	0.62	0	0.64	4.8
				Chara	−0.67	0.09	1.07			
				Baxter	1.30	0.00	2.60			
				Westonia	−0.32	0.25	0.60			
6B	162.33	163.85	Leeton	Yitpi	−0.90	0.04	1.39	0	0.57	3.6
				Chara	−0.32	0.30	0.53			
				Baxter	0.23	0.33	0.48			
				Westonia	0.94	0.03	1.51			
			Temora	Yitpi	−0.90	0.04	1.39	0	0.66	3.6
				Chara	−0.32	0.30	0.53			
				Baxter	0.23	0.33	0.48			
				Westonia	0.94	0.03	1.51			
			Yanco	Yitpi	−0.90	0.04	1.39	0	0.5	3.6
				Chara	−0.32	0.30	0.53			
				Baxter	0.23	0.33	0.48			
				Westonia	0.94	0.03	1.51			
7A	129.56	138.28	Leeton	Yitpi	1.33	0.01	1.88	0	0.9	5.9
				Chara	−0.99	0.05	1.32			
				Baxter	−0.59	0.16	0.79			
				Westonia	0.19	0.38	0.42			
			Temora	Yitpi	1.33	0.01	1.88	0	1.03	5.9
				Chara	−0.99	0.05	1.32			
				Baxter	−0.59	0.16	0.79			
				Westonia	0.19	0.38	0.42			
			Yanco	Yitpi	1.33	0.01	1.88	0	0.78	5.9
				Chara	−0.99	0.05	1.32			
				Baxter	−0.59	0.16	0.79			
				Westonia	0.19	0.38	0.42			
7B	7.17	20.89	Leeton	Yitpi	−0.75	0.12	0.93	0	1.07	3.2
				Chara	−1.10	0.06	1.20			
				Baxter	0.86	0.12	0.92			
				Westonia	0.91	0.08	1.08			
			Temora	Yitpi	−0.75	0.12	0.93	0	1.22	3.2
				Chara	−1.10	0.06	1.20			
				Baxter	0.86	0.12	0.92			
				Westonia	0.91	0.08	1.08			
			Yanco	Yitpi	−0.75	0.12	0.93	0	0.93	3.2
				Chara	−1.10	0.06	1.20			
				Baxter	0.86	0.12	0.92			
				Westonia	0.91	0.08	1.08			
Unlinked1	336.49	337	Leeton	Yitpi	0.15	0.41	0.39	0.77	0.04	0.1
				Chara	−0.18	0.39	0.41			
				Baxter	0.15	0.41	0.39			
				Westonia	−0.18	0.39	0.41			
			Temora	Yitpi	0.23	0.36	0.45	0.02	0.08	1.7
				Chara	−0.23	0.36	0.44			
				Baxter	0.23	0.36	0.45			
				Westonia	−0.23	0.36	0.44			
			Yanco	Yitpi	0.49	0.22	0.65	0.44	0.27	0.4
				Chara	−0.51	0.22	0.67			
				Baxter	0.49	0.22	0.65			
				Westonia	−0.51	0.22	0.67			

Eleven QTL were found.

The estimated polygenic or *G* × *E* covariance/correlation matrix after QTL analysis was

$$\begin{array}{l} \;\;\;\begin{array}{ccc} \text{Leeton} & \text{Temora} & \text{Yanco} \end{array}\\ \begin{array}{c} \text{Leeton}\\ \text{Temora}\\ \text{Yanco} \end{array}\left[\begin{array}{ccc} \;13.7 & \;0.87 & \;0.92\\ \;11.0 & \;11.7 & \;0.85\\ \;13.7 & \;11.6 & \;16.2 \end{array}\right] \end{array}$$

and compared to (12) we see that the genetic variances have decreased substantially (44%, 41%, and 46% for Leeton, Temora, and Yanco, respectively) after determination of putative QTL.

As a comparison univariate QTL analyses were carried out using MPWGAIM (Verbyla *et al.* 2014); see Table S3, Table S4, and Table S5. There were 11, 9, and 14 QTL detected at Leeton, Temora, and Yanco, respectively. Of the QTL detected, 4 appeared to be common to three sites and 6 were common to two of the three sites. The 4 common QTL were on 2D (2 QTL), 6B, and 7A. The univariate analyses clearly identified PPD-D1 as the major QTL influencing plant maturity across all sites. Note, however, that the size of the estimated effects is larger for the more powerful multienvironment analysis and the percentage of variance explained increases accordingly. The other QTL identified across all sites were on 2D, 6B, and 7A; the latter two turn out to be common effects across all environments in the trivariate analysis. The QTL on 2D, however, was not detected in the trivariate analysis, which is surprising, but we conjecture it is due to the forward selection of putative QTL. Interestingly 3 of the 11 QTL identified in the trivariate analysis were not identified in the univariate analyses, supporting the fact that correlation across sites offers greater power.

## Discussion

### Seed size

Of 29 QTL identified, the largest QTL for TKW was identified on chromosome 2D, consistent with a locus near PPD-D1 as reported by Williams and Sorrells (2014). The largest QTL for HW was located on 7B and appears novel. Of particular note was the number of QTL identified for both traits at the same location. Of the 29 QTL, 21 QTL were identified for both traits. However, of these 21 only 4 had the superior allele donated by the same founder for both traits. In addition, each trait had 4 independent QTL. These results, which may not be that surprising, confirm the challenges in selecting for seed morphology traits and reflect the complex genetic structure that underpins seed size and volume. The locations of the QTL identified are consistent with those in previous studies (Zhang *et al.* 2010; Gegas *et al.* 2010); in particular the QTL on 2B, 2D, 3A, and 5A are well supported in the literature; interestingly, these are the same chromosomes in this study where the founder contributing the favorable allele was the same for both traits. Conversely, the QTL on 7B for hectoliter weight explaining 14.2% of the genetic variation has not been reported previously. In addition, as in previous studies, there are a large number of genomic regions contributing significant effects for these traits, reinforcing the complex genetics underpinning these traits.

### Multienvironment trial for flowering time

The analysis detected 11 significant QTL, located on nine linkage groups. The largest QTL identified was for the QTL on 2D at the position of the PPD-D1 gene marker. This gene is one of the major genes involved in the photoperiod insensitivity to long days in wheat (Beales 2007). All sites experienced conditions that would satisfy the vernalization requirement. The major genes controlling the vernalization response in wheat are VRN1 on the group 5 chromosomes, VRN2 on 5AL, and VRN3 on 7B (Dubcovsky and Yan 2003; Trevaskis *et al.* 2003; Yan *et al.* 2003; Danyluk *et al.* 2007). The analysis detected QTL near/at these positions on 5B and 5D. As with the seed morphology data the QTL detected are well supported in the literature, in particular those on 2B, 2D, 4B, 5B, 5D, and the group 7 chromosomes (Hanocq *et al.* 2007).

### Multivariate multiparent WGAIM

The approach presented for multienvironment or multitrait analysis of MAGIC populations (MVMPWGAIM) utilizes probabilities of inheriting founder alleles for a marker or putative QTL in a whole-genome QTL analysis. A forward selection approach is used based on a likelihood-ratio test that determines whether a putative QTL should be selected and an outlier statistic is used to select the location of the putative QTL. This QTL is added to the model as a random effect for multiple traits or two random effects for multiple environments; a main effect is added in the multienvironment situation to allow for a possible common set of QTL sizes. In the latter case it is possible to test for environment-by-QTL interaction and modify the model if the hypothesis of no interaction is retained. The method allows QTL effect sizes to be determined for all founder originating alleles for each QTL and measures of strength to be specified in terms of percentage of genetic variance and a log-probability.

As with all approaches, there are advantages and disadvantages in using MVMPWGAIM. One positive was highlighted in the simulation study. The type I error rate was demonstrated to be controlled and is conservative. The power of the method is also shown to be very good. In the examples many QTL were found so effects of small QTL can be detected. The analyses of the examples took 30 and 65 hr to complete, both running on machines with 16 GB of memory. Of course these are a function of the number of QTL detected, which was large, but the biggest time factor was fitting so-called dense models. The mixed-model framework implemented accommodates trials with complex experimental designs (including spatial variation), includes derived marker/interval variables, and can include both genetic and nongenetic effects. In addition, the models used involved multiple traits or environments, which is generally computationally demanding regardless of the complexity of the model. The computational demands of fitting such a model are an issue with the asreml software used in the MVMPWAIM package or indeed with any mixed-models software that might be used. Other potential methods of analysis (for example using some kind of genome scan) may in some circumstances be less computationally and time demanding; however, they are likely to require the use of a simple linear model. This may be possible through the use of a two-stage analysis. Depending on the experimental design and model this may affect the statistical efficiency and consequently may not be ideal. The other aspect is that multivariate models are inherently difficult to fit and this is particularly true when trying to automate the process. Problems arise in the fitting of these models that sometimes require manual intervention. This means that software can be fragile and in particular very difficult to use by nonexperts.

In the power simulations, most of the QTL were found successfully. The simulation study did highlight that some QTL might be difficult to detect using multivariate methods. QTL that are expressed for a subset of traits or environments might be more difficult to detect; this is similar to the findings of Verbyla and Cullis (2012) for biparental multivariate QTL analysis and was confirmed in the power study. The effect sizes for each trait tended to be underestimated in the simulations, probably due to the small population size. Note that the number of possible scenarios for founder effect patterns and effect sizes across traits were too numerous to test. However, the simulations demonstrated that the method was able to identify QTL present only in a subset of traits. In addition, the QTL found in the two examples were large in number. As previously discussed, some of the putative QTL have been previously identified, while some QTL are novel. Overall the method performed very well across all data sets.

Multivariate methods are by their nature complex and difficult. The method presented in this article is a powerful approach for multivariate QTL analysis for MAGIC populations. The available software will allow such analysis in situations for a moderate number of traits or environments. When the number of traits/environments becomes large in number or the models are complex or have issues with convergence, it may be necessary to carry out the analyses manually or on a supercomputer. Research is underway to allow for manual intervention during the analyses, as well as further investigation of the ability of the method to identify linked QTL *vs.* pleiotropic QTL.

## Supplementary Material

Supporting Information
