# Supplementary material for: Whole-Genome Analysis of Multienvironment or Multitrait QTL in MAGIC
Source: G3 (Bethesda). 2014 Sep 1;4(9):1569–84. doi: 10.1534/g3.114.012971 (PMC4169149; doi:10.1534/g3.114.012971)
Supplement: Supporting Information [file supp_4_9_1569__index.html]

Supporting Information 

# Whole-Genome Analysis of Multienvironment or Multitrait QTL in MAGIC

## Supporting Information for Verbyla *et al.*, 2014

**Files in this Data Supplement:**

- Table S1 - MPWGAIM QTL analysis of hectolitre weight (PDF, 57 KB)
- Table S2 - MPWGAIM QTL analysis of thousand kernel weight (PDF, 57 KB)
- Table S3 - MPWGAIM analysis of flowering time for the Leeton Site (PDF, 56 KB)
- Table S4 - MPWGAIM analysis of flowering time for the Temora Site (PDF, 56 KB)
- Table S5 - MPWGAIM analysis of flowering time for the Yanco Site (PDF, 57 KB)
- File S1 - WorkedExample.R

  This file contains R code to simulate both genetic and phenotypic data and to carry out the QTL analysis of the resulting MAGIC data for a four-way cross. Both univariate and multivariate analyses are carried out. The code is self-contained in that all the necessary data is generated using R code. (.zip, 100 KB)
- File S2 - map.csv

  This file is a comma separated spreadsheet of the linkage map used in the analysis of the examples. The map has no co-located markers and is intended for QTL analysis. The spreadsheet has three columns: 1. *chr*: chromosome; 2. *marker*: marker name; 3. *distance*: genetic distance (position) for each marker on each chromosome. (.zip, 153 KB)
- File S3 - pedigree.csv

  This file is a comma separated spreadsheet containing the pedigree information of the lines in the MAGIC population used in the two examples. It has four columns 1. numeric *id*; 2. *Male* parent; 3. *Female* parent and; 4. obs which indicates the RILs that were used in trials with the number 1 with zeros elsewhere. The number id in the pedigree is just that, a number. The actual line identifiers and their correspondence to the numeric id are given in the pid.csv file. Note that the pedigree information is for the MAGIC population only. Also, the pedigree results in equal relationships across all lines so including a relationship matrix is not necessary. There is confounding of genetic effects with the overall mean effect. (.zip, 154 KB)
- File S4 - pid.csv

  A comma separated spreadsheet with two columns, 1. pid which is the pedigree id number; 2. id which is the line id of the founders and the RILs that were used in trials. This allows the pedigree information and line numbers in the data files to be associated. (.zip, 99 KB)
- File S5 - Mfounders.csv

  The founder marker scores are given in this comma separated spreadsheet. The spreadsheet has the founders listed in column 1, with markers listed in row 1 as row names. The body of the spreadsheet consists of marker scores, with bi-allelic markers having scores 0 or 1 and multi-allelic markers having their own designated possible scores. (.zip, 122 KB)
- File S6 - Mfinals.csv

  The marker scores for the RILs used in the trials are given in this comma separated spreadsheet. The first column specifies the line *id*, the first row lists the marker names and the body of the spreadsheet consists of markers scores, with bi-allelic markers having scores 0 or 1 and multi-allelic markers having their own designated possible scores. (.zip, 1.1 MB)
- File S7 - seed.csv

  The phenotypic data for the bivariate analysis for seed size traits. There are 7 columns, namely 1. *hecto*: hectolitre weight; 2. *thousKW*: thousand kernel weight; 3. *Bay*: Blocking factor in the experimental design (levels 1 to 3); 4. *Row*: row position in the two-way layout of the trial (1 to 81); 5. *Col*: column position in the two-way layout of the trial (1 to 20); 6. *pid*: the pedigree id for the founders and the RILs or four-way lines. The lines that begin with "C" in id are missing and are labelled NA.; 7. *id:* genotype or line identifier. The four-way lines begin with 'L', founders or parents grown in the trial begin with 'P', and all other lines grown in the trial begin with 'C' (for control varieties, mainly standard commercial varieties). (.zip, 121 KB)
- File S8 - zadoks.csv

  The spreadsheet for the phenotypic data for the multi-environment analysis for flowering time using zadoks score has 8 columns. They are 1. *Site*: the site of the field trial; the three sites were Leeton, Yanco and Temora; 2. *zad*: zadoks score; 3. *Block*: blocking factor in the row direction (levels 1 to 5); 4. *Row*: row position for each plot in each trial; the possible numbers vary across sites; 5. *Col*: column position for each plot in each trial; the possible numbers vary across sites; 6. *Cblock*: blocking factor across columns at Temora, with values 1 and 2; 7. *pid*: the pedigree id for the founders and the RILs or four-way lines. The lines that begin with "C" in id are missing and are labelled NA; 8. *id*: genotype or line identifier. The four-way lines begin with 'L', founders or parents grown in the trial begin with 'P', and all other lines grown in the trial begin with 'C' (for control varieties, mainly standard commercial varieties). (.zip, 137 KB)
