# Supplementary material for: Whole-Genome Analysis of Multienvironment or Multitrait QTL in MAGIC
Source: G3 (Bethesda). 2014 Sep 1;4(9):1569–84. doi: 10.1534/g3.114.012971 (PMC4169149; doi:10.1534/g3.114.012971)
Supplement: Supporting Information [file supp_4.9.1569_TableS1.pdf]

## Table S1 MPWGAIM QTL analysis of hectolitre weight

| Chromosome | dist (cM) | dist (cM) | Founder | Size | Founder Prob | Founder LOGP | Prob | % var | LOGP |
|---|---|---|---|---|---|---|---|---|---|
| 1A | 32.05 | 32.55 | Yitpi | -0.022 | 0.448 | 0.35 | 0.008 | 1.5 | 2.08 |
| | | | Chara | -0.351 | 0.023 | 1.65 | | | |
| | | | Baxter | 0.178 | 0.152 | 0.82 | | | |
| | | | Westonia | 0.179 | 0.151 | 0.82 | | | |
| 1A | 156.36 | 159.45 | Yitpi | -0.113 | 0.236 | 0.63 | 0.047 | 0.9 | 1.33 |
| | | | Chara | 0.254 | 0.046 | 1.34 | | | |
| | | | Baxter | 0.027 | 0.429 | 0.37 | | | |
| | | | Westonia | -0.179 | 0.12 | 0.92 | | | |
| 1B | 124.79 | 126.83 | Yitpi | -0.539 | 0.009 | 2.07 | 0 | 3.3 | 3.74 |
| | | | Chara | 0.301 | 0.092 | 1.04 | | | |
| | | | Baxter | 0.083 | 0.36 | 0.44 | | | |
| | | | Westonia | 0.125 | 0.288 | 0.54 | | | |
| 1D | 114.95 | 115.96 | Yitpi | -0.252 | 0.069 | 1.16 | 0.003 | 1.6 | 2.5 |
| | | | Chara | 0.033 | 0.427 | 0.37 | | | |
| | | | Baxter | -0.119 | 0.275 | 0.56 | | | |
| | | | Westonia | 0.322 | 0.036 | 1.44 | | | |
| 2A | 303.23 | 303.73 | Yitpi | 0.176 | 0.102 | 0.99 | 0.062 | 0.8 | 1.21 |
| | | | Chara | 0.036 | 0.4 | 0.4 | | | |
| | | | Baxter | -0.22 | 0.052 | 1.29 | | | |
| | | | Westonia | -0.001 | 0.499 | 0.3 | | | |
| 2B | 81.9 | 82.4 | Yitpi | 0.367 | 0.036 | 1.44 | 0.019 | 1.6 | 1.72 |
| | | | Chara | -0.291 | 0.105 | 0.98 | | | |
| | | | Baxter | -0.002 | 0.497 | 0.3 | | | |
| | | | Westonia | -0.096 | 0.321 | 0.49 | | | |
| 2B | 321.49 | 323.01 | Yitpi | 0.568 | 0.058 | 1.24 | 0 | 13.7 | 6.39 |
| | | | Chara | -0.903 | 0.015 | 1.82 | | | |
| | | | Baxter | 0.642 | 0.068 | 1.17 | | | |
| | | | Westonia | -0.425 | 0.126 | 0.9 | | | |
| 2D | 62.8 | 80.63 | Yitpi | -0.222 | 0.165 | 0.78 | 0 | 3.1 | 3.39 |
| | | | Chara | 0.494 | 0.012 | 1.93 | | | |
| | | | Baxter | -0.256 | 0.133 | 0.88 | | | |
| | | | Westonia | -0.044 | 0.423 | 0.37 | | | |
| 3B | 97.29 | 97.79 | Yitpi | 0.312 | 0.074 | 1.13 | 0.024 | 1.6 | 1.61 |
| | | | Chara | -0.107 | 0.329 | 0.48 | | | |
| | | | Baxter | -0.304 | 0.057 | 1.25 | | | |
| | | | Westonia | 0.079 | 0.371 | 0.43 | | | |
| 4B | 25.22 | 25.72 | Yitpi | 0.08 | 0.305 | 0.52 | 0.043 | 0.9 | 1.37 |
| | | | Chara | -0.167 | 0.181 | 0.74 | | | |
| | | | Baxter | 0.23 | 0.072 | 1.14 | | | |
| | | | Westonia | -0.154 | 0.174 | 0.76 | | | |
| 4D | 18 | 20.04 | Yitpi | 0.327 | 0.026 | 1.59 | 0.022 | 1.1 | 1.67 |
| | | | Chara | -0.176 | 0.138 | 0.86 | | | |
| | | | Baxter | -0.056 | 0.368 | 0.43 | | | |
| | | | Westonia | -0.109 | 0.263 | 0.58 | | | |
| 5A | 241.75 | 245.09 | Yitpi | -0.216 | 0.137 | 0.86 | 0.001 | 2.1 | 3.08 |
| | | | Chara | -0.317 | 0.05 | 1.3 | | | |
| | | | Baxter | 0.305 | 0.07 | 1.15 | | | |
| | | | Westonia | 0.205 | 0.144 | 0.84 | | | |
| 5A | 321.45 | 324.54 | Yitpi | -0.261 | 0.064 | 1.2 | 0.037 | 1.3 | 1.43 |
| | | | Chara | -0.119 | 0.274 | 0.56 | | | |
| | | | Baxter | 0.122 | 0.278 | 0.56 | | | |
| | | | Westonia | 0.243 | 0.096 | 1.02 | | | |
| 5B | 191.23 | 191.73 | Yitpi | 0.111 | 0.265 | 0.58 | 0.053 | 1.1 | 1.27 |
| | | | Chara | -0.29 | 0.058 | 1.24 | | | |
| | | | Baxter | 0.189 | 0.156 | 0.81 | | | |
| | | | Westonia | -0.025 | 0.461 | 0.34 | | | |
| 6A | 227.37 | 228.38 | Yitpi | -0.264 | 0.066 | 1.18 | 0.039 | 1.1 | 1.41 |
| | | | Chara | 0.267 | 0.097 | 1.01 | | | |
| | | | Baxter | -0.112 | 0.266 | 0.58 | | | |
| | | | Westonia | 0.093 | 0.294 | 0.53 | | | |
| 6B | 175.44 | 175.95 | Yitpi | 0.299 | 0.042 | 1.38 | 0.061 | 1.1 | 1.21 |
| | | | Chara | -0.094 | 0.329 | 0.48 | | | |
| | | | Baxter | -0.043 | 0.406 | 0.39 | | | |
| | | | Westonia | -0.176 | 0.154 | 0.81 | | | |
| 7A | 161.15 | 161.66 | Yitpi | -0.158 | 0.206 | 0.69 | 0.003 | 1.9 | 2.47 |
| | | | Chara | 0.13 | 0.247 | 0.61 | | | |
| | | | Baxter | 0.314 | 0.043 | 1.36 | | | |
| | | | Westonia | -0.305 | 0.057 | 1.24 | | | |
| 7A | 353.95 | 355.47 | Yitpi | -0.078 | 0.317 | 0.5 | 0.149 | 0.5 | 0.83 |
| | | | Chara | -0.008 | 0.478 | 0.32 | | | |
| | | | Baxter | -0.138 | 0.19 | 0.72 | | | |
| | | | Westonia | 0.216 | 0.064 | 1.19 | | | |
| 7D | 74.93 | 81.32 | Yitpi | 0.092 | 0.318 | 0.5 | 0.257 | 0.5 | 0.59 |
| | | | Chara | -0.003 | 0.494 | 0.31 | | | |
| | | | Baxter | -0.202 | 0.087 | 1.06 | | | |
| | | | Westonia | 0.104 | 0.295 | 0.53 | | | |
| Unlinked3 | 4.06 | 7.69 | Yitpi | -0.22 | 0.086 | 1.07 | 0.123 | 0.7 | 0.91 |
| | | | Chara | 0.024 | 0.454 | 0.34 | | | |
| | | | Baxter | 0.173 | 0.153 | 0.81 | | | |
| | | | Westonia | 0.011 | 0.479 | 0.32 | | | |
