# Supplementary material for: Whole-Genome Analysis of Multienvironment or Multitrait QTL in MAGIC
Source: G3 (Bethesda). 2014 Sep 1;4(9):1569–84. doi: 10.1534/g3.114.012971 (PMC4169149; doi:10.1534/g3.114.012971)
Supplement: Supporting Information [file supp_4.9.1569_TableS2.pdf]

Table S2 MPWGAIM QTL analysis of thousand kernel weight

Chromosome	dist (cM)	dist (cM)	Founder	Size	Founder Prob	Founder LOGP	Prob	% var	LOGP
2A	119.27	119.77	Yitpi	-0.463	0.117	0.93	0.002	1.5	2.72
			Chara	0.11	0.39	0.41			
			Baxter	-0.483	0.111	0.95			
			Westonia	0.744	0.028	1.55			
2B	117.41	118.42	Yitpi	1.084	0.069	1.16	0	4.7	5.33
			Chara	-1.791	0.008	2.09			
			Baxter	-0.374	0.319	0.5			
			Westonia	0.67	0.197	0.71			
2B	140.39	141.91	Yitpi	1.341	0.042	1.38	0	5.6	5.63
			Chara	-2.047	0.017	1.77			
			Baxter	0.108	0.449	0.35			
			Westonia	0.083	0.465	0.33			
2D	56.66	57.16	Yitpi	-0.682	0.054	1.27	0.004	1.6	2.4
			Chara	0.192	0.319	0.5			
			Baxter	-0.379	0.2	0.7			
			Westonia	0.759	0.04	1.4			
2D	90.57	92.09	Yitpi	-0.414	0.187	0.73	0	2.3	3.68
			Chara	0.622	0.081	1.09			
			Baxter	0.518	0.14	0.85			
			Westonia	-0.863	0.032	1.5			
2D	109.85	123.57	Yitpi	-0.32	0.317	0.5	0	5.3	4.66
			Chara	0.524	0.23	0.64			
			Baxter	-1.48	0.017	1.78			
			Westonia	0.975	0.059	1.23			
2D	165.53	166.03	Yitpi	0.392	0.264	0.58	0.015	2.6	1.82
			Chara	-0.182	0.399	0.4			
			Baxter	-1.048	0.055	1.26			
			Westonia	0.652	0.105	0.98			
3A	57.34	57.84	Yitpi	0.599	0.04	1.4	0.024	0.8	1.61
			Chara	0.035	0.461	0.34			
			Baxter	-0.25	0.241	0.62			
			Westonia	-0.447	0.1	1			
3A	246.14	246.65	Yitpi	-0.062	0.437	0.36	0.003	1.4	2.51
			Chara	-0.625	0.066	1.18			
			Baxter	-0.178	0.33	0.48			
			Westonia	0.771	0.027	1.57			
3D	39.71	145.73	Yitpi	1.888	0.046	1.34	0.005	14.2	2.34
			Chara	-1.023	0.23	0.64			
			Baxter	0.536	0.339	0.47			
			Westonia	-2.384	0.048	1.32			
5A	250.92	259.64	Yitpi	-0.825	0.023	1.64	0.001	1.7	2.95
			Chara	-0.12	0.385	0.41			
			Baxter	0.201	0.322	0.49			
			Westonia	0.641	0.054	1.26			
5A	321.45	324.54	Yitpi	-0.522	0.138	0.86	0.001	2.6	3.15
			Chara	0.045	0.467	0.33			
			Baxter	-0.734	0.096	1.02			
			Westonia	1.042	0.02	1.69			
5B	54.97	55.98	Yitpi	-0.711	0.034	1.47	0.003	1.3	2.53
			Chara	-0.109	0.389	0.41			
			Baxter	0.713	0.036	1.45			
			Westonia	0.015	0.484	0.31			
5B	360.69	361.19	Yitpi	0.93	0.016	1.8	0	1.7	3.5
			Chara	-0.449	0.21	0.68			
			Baxter	-0.428	0.176	0.75			
			Westonia	-0.177	0.346	0.46			
6B	298.64	299.15	Yitpi	-0.092	0.397	0.4	0.007	1.1	2.18
			Chara	0.289	0.229	0.64			
			Baxter	0.427	0.115	0.94			
			Westonia	-0.697	0.024	1.61			
7A	286.39	287.4	Yitpi	-0.16	0.357	0.45	0	1.9	3.61
			Chara	0.001	0.499	0.3			
			Baxter	-0.845	0.028	1.56			
			Westonia	0.876	0.024	1.61			
7B	58.9	59.4	Yitpi	0.698	0.028	1.56	0.005	1.2	2.34
			Chara	-0.586	0.059	1.23			
			Baxter	-0.056	0.441	0.36			
			Westonia	-0.135	0.355	0.45			
Unlinked3	2.02	4.06	Yitpi	-0.364	0.132	0.88	0.167	0.5	0.78
			Chara	0.01	0.49	0.31			
			Baxter	0.39	0.12	0.92			
			Westonia	-0.083	0.421	0.38			
