# Supplementary material for: Whole-Genome Analysis of Multienvironment or Multitrait QTL in MAGIC
Source: G3 (Bethesda). 2014 Sep 1;4(9):1569–84. doi: 10.1534/g3.114.012971 (PMC4169149; doi:10.1534/g3.114.012971)
Supplement: Supporting Information [file supp_4.9.1569_TableS3.pdf]

Table S3 MPWGAIM analysis of flowering time for the Leeton Site

Chromosome	Left dist (cM)	Right dist (cM)	Founder	Size	Founder Prob	Founder LOGP	Prob	% var	LOGP
2B	81.39	81.9	Yitpi	-0.58	0.192	0.72	0.001	1.3	2.9
			Chara	-1.171	0.062	1.21			
			Baxter	0.357	0.308	0.51			
			Westonia	1.27	0.03	1.52			
2D	7.35	31.25	Yitpi	-4.648	0.004	2.42	0	49.5	52.66
			Chara	4.068	0.017	1.78			
			Baxter	-6.409	0	3.73			
			Westonia	4.438	0.007	2.16			
2D	56.66	57.16	Yitpi	-1.262	0.015	1.83	0.002	1.1	2.69
			Chara	0.63	0.136	0.86			
			Baxter	0.49	0.212	0.67			
			Westonia	0.058	0.462	0.34			
2D	136.19	137.2	Yitpi	0.557	0.147	0.83	0.007	0.8	2.17
			Chara	0.292	0.3	0.52			
			Baxter	-1.05	0.019	1.72			
			Westonia	0.142	0.389	0.41			
3D	39.71	145.73	Yitpi	2.352	0.07	1.16	0.012	9.7	1.93
			Chara	-1.292	0.253	0.6			
			Baxter	1.358	0.228	0.64			
			Westonia	-3.161	0.057	1.25			
4B	96.06	96.56	Yitpi	0.136	0.41	0.39	0	1.5	4.09
			Chara	0.412	0.264	0.58			
			Baxter	0.805	0.098	1.01			
			Westonia	-1.456	0.009	2.05			
5A	310.18	310.68	Yitpi	-0.39	0.204	0.69	0.013	0.7	1.9
			Chara	0.166	0.367	0.44			
			Baxter	-0.669	0.093	1.03			
			Westonia	0.841	0.038	1.42			
5B	240.97	241.47	Yitpi	0.646	0.1	1	0.003	0.8	2.47
			Chara	-1.066	0.019	1.72			
			Baxter	0.313	0.28	0.55			
			Westonia	0.044	0.468	0.33			
6B	149.9	162.33	Yitpi	-0.986	0.099	1.01	0	2.1	4.37
			Chara	-1.128	0.079	1.1			
			Baxter	0.588	0.211	0.68			
			Westonia	1.371	0.029	1.54			
7A	125.93	129.56	Yitpi	0.829	0.059	1.23	0.003	1	2.47
			Chara	-0.457	0.194	0.71			
			Baxter	-0.88	0.048	1.32			
			Westonia	0.44	0.205	0.69			
7B	44.37	44.88	Yitpi	-0.166	0.37	0.43	0.006	0.8	2.21
			Chara	-0.92	0.029	1.54			
			Baxter	0.389	0.221	0.66			
			Westonia	0.642	0.092	1.04			
