# Supplementary material for: Whole-Genome Analysis of Multienvironment or Multitrait QTL in MAGIC
Source: G3 (Bethesda). 2014 Sep 1;4(9):1569–84. doi: 10.1534/g3.114.012971 (PMC4169149; doi:10.1534/g3.114.012971)
Supplement: Supporting Information [file supp_4.9.1569_TableS4.pdf]

Table S4 MPWGAIM analysis of flowering time for the Temora Site

Chromosome	Left dist (cM)	Right dist (cM)	Founder	Size	Founder Prob	Founder LOGP	Prob	% var	LOGP
2D	7.35	31.25	Yitpi	-2.931	0.008	2.12	0	49.3	40.64
			Chara	3.121	0.013	1.88			
			Baxter	-5.384	0	4.9			
			Westonia	3.767	0.002	2.77			
2D	56.66	57.16	Yitpi	-1.702	0.005	2.32	0	2.9	5.79
			Chara	0.744	0.126	0.9			
			Baxter	0.604	0.19	0.72			
			Westonia	0.236	0.365	0.44			
2D	137.2	138.21	Yitpi	0.481	0.167	0.78	0.003	1.1	2.48
			Chara	0.423	0.208	0.68			
			Baxter	-0.999	0.017	1.76			
			Westonia	0.044	0.462	0.34			
5D	64.86	68.49	Yitpi	-0.236	0.318	0.5	0.001	1.4	3.3
			Chara	-0.694	0.08	1.1			
			Baxter	1.019	0.015	1.83			
			Westonia	-0.143	0.386	0.41			
6B	162.33	163.85	Yitpi	-0.975	0.034	1.47	0.001	1.6	3.03
			Chara	-0.201	0.372	0.43			
			Baxter	0.201	0.356	0.45			
			Westonia	0.911	0.039	1.41			
6D	140.01	151.17	Yitpi	0.506	0.184	0.73	0.012	1	1.91
			Chara	-0.841	0.028	1.55			
			Baxter	0.191	0.342	0.47			
			Westonia	0.1	0.434	0.36			
7A	125.93	129.56	Yitpi	1.371	0.011	1.94	0	2.5	5.28
			Chara	-0.833	0.083	1.08			
			Baxter	-0.776	0.098	1.01			
			Westonia	0.14	0.408	0.39			
7D	111.1	111.6	Yitpi	-0.098	0.415	0.38	0.059	0.6	1.23
			Chara	-0.702	0.044	1.36			
			Baxter	0.455	0.14	0.85			
			Westonia	0.313	0.246	0.61			
