# Supplementary material for: Whole-Genome Analysis of Multienvironment or Multitrait QTL in MAGIC
Source: G3 (Bethesda). 2014 Sep 1;4(9):1569–84. doi: 10.1534/g3.114.012971 (PMC4169149; doi:10.1534/g3.114.012971)
Supplement: Supporting Information [file supp_4.9.1569_TableS5.pdf]

## Table S5 MPWGAIM analysis of flowering time for the Yanco Site

| Chromosome | Left dist (cM) | Right dist (cM) | Founder | Size | Founder Prob | Founder LOGP | Prob | % var | LOGP |
|---|---|---|---|---|---|---|---|---|---|
| 2A | 26.48 | 26.98 | Yitpi | -0.332 | 0.224 | 0.65 | 0.077 | 0.3 | 1.11 |
| | | | Chara | 0.034 | 0.472 | 0.33 | | | |
| | | | Baxter | -0.345 | 0.216 | 0.67 | | | |
| | | | Westonia | 0.608 | 0.062 | 1.21 | | | |
| 2B | 82.4 | 88.23 | Yitpi | -0.512 | 0.297 | 0.53 | 0 | 2.9 | 5.75 |
| | | | Chara | -2.372 | 0.012 | 1.94 | | | |
| | | | Baxter | 0.885 | 0.186 | 0.73 | | | |
| | | | Westonia | 1.656 | 0.043 | 1.36 | | | |
| 2D | 7.35 | 31.25 | Yitpi | -4.902 | 0.003 | 2.5 | 0 | 60 | 82.48 |
| | | | Chara | 4.4 | 0.012 | 1.93 | | | |
| | | | Baxter | -9.16 | 0 | 6.42 | | | |
| | | | Westonia | 5.304 | 0.002 | 2.68 | | | |
| 2D | 56.66 | 57.16 | Yitpi | -1.121 | 0.022 | 1.66 | 0.003 | 0.8 | 2.48 |
| | | | Chara | 0.716 | 0.096 | 1.02 | | | |
| | | | Baxter | 0.447 | 0.222 | 0.65 | | | |
| | | | Westonia | -0.127 | 0.415 | 0.38 | | | |
| 3D | 149.76 | 151.28 | Yitpi | 0.838 | 0.081 | 1.09 | 0.036 | 0.4 | 1.44 |
| | | | Chara | -0.294 | 0.385 | 0.41 | | | |
| | | | Baxter | -0.299 | 0.383 | 0.42 | | | |
| | | | Westonia | -0.358 | 0.361 | 0.44 | | | |
| 4B | 75.62 | 77.66 | Yitpi | -0.28 | 0.271 | 0.57 | 0.011 | 0.5 | 1.95 |
| | | | Chara | 0.495 | 0.158 | 0.8 | | | |
| | | | Baxter | 0.553 | 0.128 | 0.89 | | | |
| | | | Westonia | -0.825 | 0.038 | 1.42 | | | |
| 5A | 199.63 | 203.26 | Yitpi | -0.612 | 0.118 | 0.93 | 0.002 | 0.7 | 2.77 |
| | | | Chara | 0.962 | 0.027 | 1.57 | | | |
| | | | Baxter | -0.597 | 0.136 | 0.87 | | | |
| | | | Westonia | 0.176 | 0.363 | 0.44 | | | |
| 5B | 240.97 | 241.47 | Yitpi | 0.292 | 0.312 | 0.51 | 0 | 1 | 3.86 |
| | | | Chara | -1.336 | 0.013 | 1.87 | | | |
| | | | Baxter | -0.063 | 0.46 | 0.34 | | | |
| | | | Westonia | 0.994 | 0.058 | 1.24 | | | |
| 5D | 64.86 | 68.49 | Yitpi | -0.537 | 0.208 | 0.68 | 0 | 1.6 | 6.2 |
| | | | Chara | -0.882 | 0.089 | 1.05 | | | |
| | | | Baxter | 1.534 | 0.007 | 2.13 | | | |
| | | | Westonia | -0.248 | 0.352 | 0.45 | | | |
| 6B | 35.09 | 35.59 | Yitpi | -0.127 | 0.379 | 0.42 | 0.101 | 0.3 | 0.99 |
| | | | Chara | -0.47 | 0.119 | 0.92 | | | |
| | | | Baxter | 0.503 | 0.088 | 1.05 | | | |
| | | | Westonia | 0.062 | 0.449 | 0.35 | | | |
| 6B | 162.33 | 163.85 | Yitpi | -0.647 | 0.151 | 0.82 | 0.001 | 1 | 2.96 |
| | | | Chara | -0.951 | 0.094 | 1.03 | | | |
| | | | Baxter | 0.33 | 0.304 | 0.52 | | | |
| | | | Westonia | 1.153 | 0.029 | 1.53 | | | |
| 7A | 129.56 | 138.28 | Yitpi | 1.854 | 0.007 | 2.13 | 0 | 2.1 | 7.03 |
| | | | Chara | -0.878 | 0.123 | 0.91 | | | |
| | | | Baxter | -1.08 | 0.079 | 1.1 | | | |
| | | | Westonia | -0.094 | 0.452 | 0.35 | | | |
| 7B | 101.37 | 102.38 | Yitpi | 0.808 | 0.039 | 1.41 | 0.032 | 0.4 | 1.5 |
| | | | Chara | -0.257 | 0.329 | 0.48 | | | |
| | | | Baxter | -0.07 | 0.445 | 0.35 | | | |
| | | | Westonia | -0.534 | 0.125 | 0.9 | | | |
| Unlinked1 | 5.08 | 5.58 | Yitpi | 0.568 | 0.302 | 0.52 | 0.01 | 0.6 | 2.01 |
| | | | Chara | -0.649 | 0.277 | 0.56 | | | |
| | | | Baxter | 0.568 | 0.302 | 0.52 | | | |
| | | | Westonia | -0.649 | 0.277 | 0.56 | | | |
