# Supplementary material for: Whole-Genome Analysis of Multienvironment or Multitrait QTL in MAGIC
Source: G3 (Bethesda). 2014 Sep 1;4(9):1569–84. doi: 10.1534/g3.114.012971 (PMC4169149; doi:10.1534/g3.114.012971)
Supplement: Supporting Information [file supp_4.9.1569_FileS1.zip › FileS1/READ_ME.pdf]

File S1

WorkedExample.R

File S1 is available for download as a text file (with the extension .R to be used in the R environment) at http://www.g3journal.org/lookup/suppl/doi:10.1534/g3.114.012971/-/DC1

This file contains R code to simulate both genetic and phenotypic data and to carry out the QTL analysis of the resulting MAGIC data for a four-way cross. Both univariate and multivariate analyses are carried out. The code is self-contained in that all the necessary data is generated using R code.
