# Supplementary material for: Whole-Genome Analysis of Multienvironment or Multitrait QTL in MAGIC
Source: G3 (Bethesda). 2014 Sep 1;4(9):1569–84. doi: 10.1534/g3.114.012971 (PMC4169149; doi:10.1534/g3.114.012971)
Supplement: Supporting Information [file supp_4.9.1569_FileS2.zip › FileS2/READ_ME.pdf]

File S2

map.csv

File S2 is available for download as a comma separated csv file at

http://www.g3journal.org/lookup/suppl/doi:10.1534/g3.114.012971/-/DC1

This file is a comma separated spredsheet of the linkage map used in the analysis of the examples. The map has no co-located markers and is intended for QTL analysis. The spreadsheet has three columns:

1. *chr*: chromosome
2. *marker*: marker name
3. *distance*: genetic distance (position) for each marker on each chromosome
