# Supplementary material for: Whole-Genome Analysis of Multienvironment or Multitrait QTL in MAGIC
Source: G3 (Bethesda). 2014 Sep 1;4(9):1569–84. doi: 10.1534/g3.114.012971 (PMC4169149; doi:10.1534/g3.114.012971)
Supplement: Supporting Information [file supp_4.9.1569_FileS3.zip › FileS3/READ_ME.pdf]

File S3

pedigree.csv

File S3 is available for download as a comma separated csv file at

http://www.g3journal.org/lookup/suppl/doi:10.1534/g3.114.012971/-/DC1

This file is a comma separated spreadsheet containing the pedigree information of the lines in the MAGIC population used in the two examples. It has four columns

1. numeric *id*,
2. *Male* parent,
3. *Female* parent and
4. *obs* which indicates the RILs that were used in trials with the number 1 with zeros elsewhere.

The number *id* in the pedigree is just that, a number. The actual line identifiers and their correspondence to the numeric *id* are given in the `pid.csv` file.

Note that the pedigree information is for the MAGIC population only. Also, the pedigree results in equal relationships across all lines so including a relationship matrix is not necessary. There is confounding of genetic effects with the overall mean effect.
