# Supplementary material for: Whole-Genome Analysis of Multienvironment or Multitrait QTL in MAGIC
Source: G3 (Bethesda). 2014 Sep 1;4(9):1569–84. doi: 10.1534/g3.114.012971 (PMC4169149; doi:10.1534/g3.114.012971)
Supplement: Supporting Information [file supp_4.9.1569_FileS4.zip › FileS4/READ_ME.pdf]

**File S4**

**pid.csv**

File S4 is available for download as a comma separated csv file at

http://www.g3journal.org/lookup/suppl/doi:10.1534/g3.114.012971/-/DC1

A comma separated spreadsheet with two columns,

1. *pid* which is the *p*edigree *id* number

2. *id* which is the line *id* of the founders and the RILs that were used in trials.

This allows the pedigree information and line numbers in the data files to be associated.
