# Supplementary material for: Whole-Genome Analysis of Multienvironment or Multitrait QTL in MAGIC
Source: G3 (Bethesda). 2014 Sep 1;4(9):1569–84. doi: 10.1534/g3.114.012971 (PMC4169149; doi:10.1534/g3.114.012971)
Supplement: Supporting Information [file supp_4.9.1569_FileS5.zip › FileS5/READ_ME.pdf]

File S5

Mfounders.csv

File S5 is available for download as a comma separated csv file at

http://www.g3journal.org/lookup/suppl/doi:10.1534/g3.114.012971/-/DC1

The founder marker scores are given in this comma separated spreadsheet. The spreadsheet has the founders listed in column 1, with markers listed in row 1 as row names. The body of the spreadsheet consists of marker scores, with bi-allelic markers having scores 0 or 1 and multi-allelic markers having their own designated possible scores.
