# Supplementary material for: Whole-Genome Analysis of Multienvironment or Multitrait QTL in MAGIC
Source: G3 (Bethesda). 2014 Sep 1;4(9):1569–84. doi: 10.1534/g3.114.012971 (PMC4169149; doi:10.1534/g3.114.012971)
Supplement: Supporting Information [file supp_4.9.1569_FileS6.zip › FileS6/READ_ME.pdf]

File S6

Mfinals.csv

File S6 is available for download as a comma separated csv file at

http://www.g3journal.org/lookup/suppl/doi:10.1534/g3.114.012971/-/DC1

The marker scores for the RILs used in the trials are given in this comma separated spreadsheet. The first column specifies the line *id*, the first row lists the marker names and the body of the spreadsheet consists of markers scores, with bi-allelic markers having scores 0 or 1 and multi-allelic markers having their own designated possible scores.
