# Supplementary material for: Whole-Genome Analysis of Multienvironment or Multitrait QTL in MAGIC
Source: G3 (Bethesda). 2014 Sep 1;4(9):1569–84. doi: 10.1534/g3.114.012971 (PMC4169149; doi:10.1534/g3.114.012971)
Supplement: Supporting Information [file supp_4.9.1569_FileS7.zip › FileS7/READ_ME.pdf]

File S7

seed.csv

File S7 is available for download as a comma separated csv file at

http://www.g3journal.org/lookup/suppl/doi:10.1534/g3.114.012971/-/DC1

The phenotypic data for the bivariate analysis for seed size traits. There are 7 columns, namely

1. *hecto*: hectolitre weight
2. *thousKW*: thousand kernel weight
3. *Bay*: Blocking factor in the experimental design (levels 1 to 3)
4. *Row*: row position in the two-way layout of the trial (1 to 81)
5. *Col*: column position in the two-way layout of the trial (1 to 20)
6. *pid*: the pedigree id for the founders and the RILs or four-way lines. The lines that begin with ``C'' in *id* are missing and are labelled `NA`.
7. *id*: genotype or line identifier. The four-way lines begin with `L', founders or parents grown in the trial begin with `P', and all other lines grown in the trial begin with `C' (for control varieties, mainly standard commercial varieties).
