# Supplementary material for: Whole-Genome Analysis of Multienvironment or Multitrait QTL in MAGIC
Source: G3 (Bethesda). 2014 Sep 1;4(9):1569–84. doi: 10.1534/g3.114.012971 (PMC4169149; doi:10.1534/g3.114.012971)
Supplement: Supporting Information [file supp_4.9.1569_FileS8.zip › FileS8/READ_ME.pdf]

File S8

zadoks.csv

File S8 is available for download as a comma separated csv file at

http://www.g3journal.org/lookup/suppl/doi:10.1534/g3.114.012971/-/DC1

The spreadsheet for the phenotypic data for the multi-environment analysis for flowering time using zadoks score has 8 columns. They are

1. *Site*: the site of the field trial; the three sites were Leeton, Yanco and Temora.
2. *zad*: zadoks score
3. *Block*: blocking factor in the row direction (levels 1 to 5)
4. *Row*: row position for each plot in each trial; the possible numbers vary across sites.
5. *Col*: column position for each plot in each trial; the possible numbers vary across sites.
6. *Cblock*: blocking factor across columns at Temora, with values 1 and 2.
7. *pid*: the pedigree id for the founders and the RILs or four-way lines. The lines that begin with ``C'' in *id* are missing and are labelled `NA`.
8. *id*: genotype or line identifier. The four-way lines begin with `L', founders or parents grown in the trial begin with `P', and all other lines grown in the trial begin with `C' (for control varieties, mainly standard commercial varieties).
